# Reviewer Acknowledgement BMC Public Health

**DOI:** 10.1186/1471-2458-13-223

**Published:** 2013-04-12

**Authors:** Natalie Pafitis

**Affiliations:** 1 BioMed Central, 236 Gray’s Inn Road, London, WC1X 8HB, UK

## Abstract

**Contributing reviewers:**

The editors of BMC Public Health would like to thank all our reviewers who have contributed to the journal in 2012.

## 

Mette Aadahl

Denmark

Kristal Aaron

United States of America

Randi W Aas

Norway

Preben Aavitsland

Norway

Azriani Ab Rahman

Malaysia

**Magda Abd El**-**Salam**

Egypt

Mohamed Abdel-Hamid

Egypt

Mohamed Hatha Abdulla

India

Khatijah Abdullah

Malaysia

Asnawi Abdullah

Indonesia

Dawit Shawel Abebe

Norway

Gemeda Abebe

Ethiopia

Thomas Abel

Switzerland

Sophie Abgrall

France

Rachel T. Abraham

United States of America

Joshua Abrams

United States of America

Sandra Abreu

Portugal

Karim Abu-Omar

Germany

Gabriele Accetta

Italy

Stefan Acosta

Sweden

Anna Adachi-Mejia

United States of America

Philippe Adam

Australia

James Adams

United States of America

Jean Adams

United Kingdom

Ayo Adebowale

Nigeria

Ramesh Adhikari

Nepal

Shiva Adhikari

Nepal

Sam Adjei

Ghana

Philip Adongo

Ghana

Angeles Aedo

Mexico

Patricia Agaba

Nigeria

Suneth Agampodi

Sri Lanka

Gabriel Agboado

United Kingdom

Calypse Bessem Agborsangaya

Canada

Saad Agha

Iraq

Kingsley Agho

Australia

Sutapa Agrawal

India

Andrés A. Agudelo-Suárez

Spain

Charles Agyemang

Netherlands

Nibras Ahmed

Norway

Brian Ahmedani

United States of America

Kamran Mahmood Ahmed Aziz

Saudi Arabia

Marion C Aichberger

Germany

Allison Aiello

United States of America

Barbara Ainsworth

United States of America

Elaheh Ainy

Iran

Luísa Aires

Portugal

Ademola Ajuwon

Nigeria

Gamze Akbulut

Turkey

Adenike Akhigbe

Nigeria

Dora Olufunmilola Akinboye

Nigeria

Rachael Adeyanju Akinola

Nigeria

Manas Akmatov

Germany

Ersin Akpinar

Turkey

Susan Albrecht

United States of America

Jennifer Albrecht

United States of America

Jeanine Albu

United States of America

Philip Alcabes

United States of America

Alessandra Alciati

Italy

Samim Al-Dabbagh

Iraq

Alicia Aleman

Uruguay

Farrokh Alemi

United States of America

Fekadu Mazengia Alemu

Sudan

Airlane Alencar

Brazil

Lucienne Alencar

Brazil

Murray Alexander

Canada

Krystallenia Alexandraki

Greece

Ute Alexy

Germany

Hazzaa Al-Hazzaa

Saudi Arabia

Jerilyn Allen

United States of America

Naomi Allen

United Kingdom

Michele Allen

United States of America

Franz Allerberger

Austria

Farzana Alli

South Africa

Monica Alonso Gonzalez

United States of America

Hillel Alpert

United States of America

Astrid Altena

Netherlands

Benjamin Althouse

United States of America

Laura C Altobelli

Peru

Alberto Alves

Portugal

Reza Amani

Iran

Hemantha Amarasinghe

Sri Lanka

Alemayehu Amberbir

Ethiopia

Nayyereh Aminisani

Australia

Amanda Amos

United Kingdom

Eugenia Amporfu

Ghana

Panagiotis Anagnostis

Greece

Raghupathy Anchala

United Kingdom

Laura Anderson

Canada

Laura Anderson

United Kingdom

Vitas Anderson

Australia

Claire Anderson

United Kingdom

Stuart Anderson

United Kingdom

Brooke Anderson

United States of America

Tatiana Andreeva

Ukraine

Eleni Andreou

Greece

Richard Andrews

United States of America

Armelle Andro

France

Aranka Anema

Canada

Philip Anglewicz

United States of America

Luis Anibarro

Spain

Abebe Animut

Ethiopia

Luciana Anselmi

Brazil

Fernando Antonanzas

Spain

Georgios Antonogeorgos

Greece

Samuel Anya

Gambia

Virginia Aparicio

Spain

Kaija Appelqvist-Schmidlechner

Finland

Christine Arcari

United States of America

Norm Archer

Canada

Chris Ardern

Canada

Ella Arensman

Ireland

Matthew Arentz

United States of America

Kristopher Arheart

United States of America

Miranda Elaine Glynis Armstrong

United Kingdom

Greg Armstrong

Australia

Rebecca Armstrong

Australia

Monika Arora

India

Amit Arora

Australia

Wouter Arrazola de Onate

Belgium

Manuel Arroyo-Morales

Spain

Antonio Ascensao

Portugal

Ann Aschengrau

United States of America

Ali Asgary

Canada

Bryndis Bjork Asgeirsdottir

Iceland

Wes Ashford

United States of America

Abdelatif Ashmaig

Sudan

Kingsley Asiedu

Switzerland

Ali Assabri

Yemen

Yibeltal Assefa

Ethiopia

Nega Assefa

Ethiopia

Thomas Astell-Burt

United Kingdom

Olayinka Atilola

Nigeria

Andy Atkin

United Kingdom

Evan Atlantis

Australia

Evan Atlantis

Australia

Suzanne Audrey

United Kingdom

Robert Aunger

United Kingdom

Astrid Austvoll-Dahlgren

Norway

Roseanne Autran

Portugal

Susan Averett

United States of America

Makoto Ayabe

Japan

Touraj Ayazi

Norway

John Ayisi

Kenya

Mohamed Ayoya

United States of America

Leila Azadbakht

Iran

Sunday Azagba

Canada

Kenichi Azuma

Japan

Stella Babalola

United States of America

Juliet Babirye

Uganda

Valerio Bacak

United States of America

Abdulgafoor Bachani

United States of America

Kathryn Backholer

Australia

Alaa Badawi

Canada

Jong-Myon Bae

Korea South

Cristina Baena

Netherlands

Giovanni Baglio

Italy

Helen Bailey

France

Ross Bailie

Australia

Graham Baker

United Kingdom

Dean Baker

United States of America

Tim Baker

United States of America

Jan Vilhelm Bakke

Norway

Dimitrios Balatsouras

Greece

Kylie Ball

Australia

Terri Ballard

Italy

Montse Ballbe

Spain

James Balmford

Australia

Danilo Baltieri

Brazil

Maria Baltzer

Sweden

Clare Bambra

United Kingdom

James Bamidele

Nigeria

Matthew (Mateo) Banegas

United States of America

Wei Bao

United States of America

Lili Bao

United States of America

Fátima Baptista

Portugal

Bridget Barber

Australia

Maria Barcelo

Spain

Ilaria Barchetta

Italy

Alan Barclay

Australia

Barbara Bardenheier

United States of America

Jan Barendregt

Australia

Charles Bark

United States of America

Jane Barlow

United Kingdom

Fiona Kate Barlow

Australia

Sivia Barnoy

Israel

Briana Barocas

United States of America

Helen Barrett

Australia

Francoise Barten

Netherlands

Yvette Bartholomee

Netherlands

Mel Bartley

United Kingdom

Kidist Bartolomeos

Switzerland

Xavier Basagana

Spain

Sajid Bashir

Pakistan

Paulin Basinga

Rwanda

Abdul Basit

Pakistan

Tibor Baska

Slovakia

Moses Bateganya

Uganda

Anwar Batieha

Jordan

Antoine Baumann

France

Steven Baumrucker

United States of America

Iacopo Baussano

United Kingdom

Himmatrao Bawaskar

India

Grzegorz Bazylak

Poland

J. David Beatty

United States of America

Alison Beauchamp

Australia

Annette Beautrais

United States of America

Nam Seon Beck

Korea South

Anders Beckman

Sweden

Raman Bedi

United Kingdom

Marin Been

Netherlands

Janice Bell

United States of America

Robin Bell

Australia

Nathaniel Bell

Canada

Temitope Bello

Nigeria

Folasade Bello

Nigeria

Eran Bendavid

United States of America

Robert Bendel

United States of America

Kofi Benefo

United States of America

Abdulbari Bener

Qatar

Corina Benjet

Mexico

Isabela Bensenor

Brazil

Michaela Benzeval

United Kingdom

Bayeha Bera

Ethiopia

Andrea Beratarrechea

Argentina

Janneke Berecki-Gisolf

Australia

Rigmor Berg

Norway

John E. Berg

Norway

Gretchen Berggren

United States of America

Natasa Berginc

Slovenia

David Berrigan

United States of America

Helen Louise Berry

Australia

Helen Berry

Australia

Tanya Berry

Canada

Mael Bessaud

France

Jolly Beyeza-Kashesya

Uganda

Caryl M Beynon

United Kingdom

Chris Beyrer

United States of America

Gajananda Bhandari

Nepal

Ruchi Bhandari

United States of America

Shalini Bharat

India

Premananda Bharati

India

Sujit Bhattacharya

India

Raj Bhopal

United Kingdom

Zulfiqar Bhutta

Pakistan

Ettore Bidoli

Italy

Jade Bilardi

Australia

Martha Billings

United States of America

Henry Binder

United States of America

Colin Binns

Australia

Lauren Birks

Canada

Gallus Bischof

Germany

Alison Bish

United Kingdom

Zeno Bisoffi

Italy

Marcio Sommer Bittencourt

United States of America

Danielle Bivanco de Lima

Brazil

Jonas Björk

Sweden

Gunnar Aksel Bjune

Norway

Robert Black

United States of America

Andrew Blann

United Kingdom

Evan Blecher

United States of America

Chantal Bleeker-Rovers

Netherlands

Sara Bleich

United States of America

Mats Blid

Sweden

Ilse Blignault

Australia

Victoria Blinder

United States of America

Daniel Blockmans

Belgium

Laura Blue

United States of America

Ian Boardley

United Kingdom

Natalia Bobrova

United Kingdom

Delia Boccia

United Kingdom

Stefania Boccia

Italy

Walter Bockting

United States of America

Joseph Boden

New Zealand

J. Ties Boerma

Switzerland

Henrik Bøggild

Denmark

Richard Bohannon

United States of America

Vanessa Bolejack

United States of America

Kristy Bolton

Australia

Andrea Bombak

Canada

Jens Peter Bonde

Denmark

Susan Bondy

Canada

Catriona Bonfiglioli

Australia

Stefanos Bonovas

Greece

Claudio Bontempi

Italy

Patcharee Boonsiri

Thailand

Alison Booth

Australia

Robert Booth

United States of America

Robert Booy

Australia

Matthias Borchert

Germany

Theda Borde

Germany

Johan Borg

Sweden

Carolina Borges

Brazil

Isabelle Borget

France

Vanni Borghi

Italy

Piero Borgia

Italy

Afshin Borhani Haghighi

Iran

Sonia Borrell

Switzerland

Luisa Borrell

United States of America

Xavier Bosch-Capblanch

Switzerland

Cristina Bosetti

Italy

Hans Bosma

Netherlands

Robert Bossarte

United States of America

Ercolie Bossema

Netherlands

Georgiana Bostean

United States of America

Jérémie Botton

France

Barbara J Boucher

United Kingdom

Frederic Bouder

Netherlands

Anne-Déborah Bouhnik

France

Arnaud Bourdin

France

Deborah Bowen

United States of America

Emma Boyland

United Kingdom

Raymond Boyle

United States of America

Marek Brabec

Czech Republic

Kristina Brache

Canada

Robert Bradley

United States of America

Robert Bradley

United States of America

Anke Bramesfeld

Germany

Jenny Brands

Australia

Kathryn Braun

United States of America

Manuel Bravo

Spain

Hans Johan Breidablik

Norway

Nancy Brener

United States of America

Lisa Brenner

United States of America

Joan Brewster

Canada

Philip Brickner

United States of America

Julie Brimblecombe

Australia

Helena Britt

Australia

Jonathan Broadbent

New Zealand

Robert Brooks

Australia

Leonie S Brose

United Kingdom

Sandra Brouwer

Netherlands

Evelien Brouwers

Netherlands

Shawn Brown

United States of America

Elizabeth Brown

United States of America

Alison Brown

United Kingdom

Sally Brown

United Kingdom

Stephen Brown

United Kingdom

Jessica Browne

Australia

Johannes Brug

Netherlands

Andre Brunoni

Brazil

Irene Brüske

Germany

Linda Bryant

New Zealand

Jamie Bryant

Australia

Jennifer Bryce

United States of America

Patrick Brzoska

Germany

Udo Buchholz

Germany

William Buchta

United States of America

Jennifer Buckley

Australia

Christine Budke

United States of America

James Buehler

United States of America

Mohammed Bukar

Nigeria

Kanitta Bundhamcharoen

Thailand

Tim Bungum

United States of America

Rebecca Bunnell

Kenya

Alex Burdorf

Netherlands

Esther Buregyeya

Uganda

Pierre-Regis Burgel

France

Diana Burgess

United States of America

Nathalie Burkert

Austria

Gregor Burkhart

Portugal

Frederick Burkle

United States of America

Frederick Burkle

United States of America

Stephanie Burrows

Canada

Nancy Busen

United States of America

Luciana Butini Oliveira

Brazil

Frances Butterfoss

United States of America

Anne Buve

Belgium

Francis Bwambale

Uganda

Peter Byass

Sweden

Carol Byrd-Bredbenner

United States of America

Xiaodong Cai

United States of America

Demetria Cain

United States of America

Banu Cakir

Turkey

Brent Caldwell

New Zealand

Erica Caldwell

Australia

Gillian Callaghan

United Kingdom

Cynthia Callard

Canada

Catherine Calvin

United Kingdom

Lisa Calvocoressi

United States of America

Vitaliano Cama

United States of America

Carlos Camargo

United States of America

Emmanuelle Cambois

France

Dexter Canoy

United Kingdom

Joseph Canterino

United States of America

Xavier Carcopino

France

Rachel Carey

Australia

Waldemar (Wally) Carlo

United States of America

Terry Carney

Australia

Jean Caron

Canada

L Rand Carpenter

United States of America

David Carpenter

United States of America

Luis Roman Carrasco

Singapore

Giulia Carreras

Italy

Guillermo Carroli

Argentina

Tom Carroll

Australia

Dean Carson

Australia

Marissa Carter

United States of America

Owen Carter

Australia

Tim Carter

United Kingdom

Filomena Marino Carvalho

Brazil

Celso R F Carvalho

Brazil

Maria Joana Carvalho

Portugal

Martí Casals Toquero

Spain

Robert Casson

Australia

Carlos Castañeda-Orjuela

Colombia

Antoni Castel

Spain

Jesús Castilla

Spain

Amparo Castillo

United States of America

Carlos Castillo-Duran

Chile

Carla Castillo-Laborde

Chile

Jose Castro

United States of America

Ferrán Catalá-López

Spain

Lucrezia Catania

Italy

Dolores Catelan

Italy

Willard Cates

United States of America

Andy Catley

Ethiopia

Mauro Ceccanti

Italy

Anna Cejka

United States of America

Rodrigo Cerda

United States of America

Ester Cerin

Hong Kong

Betty Chaar

Australia

Henri Chabrol

France

Michael Chaiton

Canada

Surasak Chaiyasong

Thailand

Apu Chakraborty

Canada

Wilfred Chalamira-Nkhoma

Zimbabwe

Patricia Chalela

United States of America

Eric Chamot

United States of America

Juliana Chan

China

Keith KW Chan

Hong Kong

Sally Chan

Singapore

Frank Chan

Hong Kong

Subhash Chandir

United States of America

Daniel Chandler

United Kingdom

Pascal Chanez

France

Shu-Sen Chang

Hong Kong

Wei-Ching Chang

Canada

Chin-Kuo Chang

United Kingdom

Chun Chang

China

Po-Yuan Chang

Taiwan

Ubon Cha’on

Thailand

Steven Chapman

United States of America

Richard Chapman

United States of America

Arundhati Char

India

Claire Chase

United States of America

Nishi Chaturvedi

United Kingdom

Sanjay Chaturvedi

India

Nearkasen Chau

France

Wisit Chaveepojnkamjorn

Thailand

Julius Cheah

Malaysia

Chunming Chen

China

Guanmin Chen

Canada

Huei-Yang Chen

Australia

Ying-Yeh Chen

Taiwan

Yueh-Chih Chen

Taiwan

Shih-Jen Chen

Taiwan

Shawn Chen

Taiwan

Xiang-Sheng Chen

China

Zhuo Chen

United States of America

Jiaxu Chen

China

John Cherrie

United Kingdom

Terusha Chetty

South Africa

Emmanuelle Chevallier-Portalez

France

Rosy Chhabra

United States of America

Pragti Chhabra

India

Donald Chi

United States of America

Carlos Chiatti

Italy

Carla Chibwesha

Zambia

Li-Yin Chien

Taiwan

Francesco Chini

Italy

Jean-Philippe Chippaux

France

Maureen Chileshe Chisembele

Zambia

Yueh-Hsiu Mathilda Chiu

United States of America

Hong-Jun Cho

Korea South

Eo Rin Cho

Korea South

Gabriel Chodick

Israel

Edward Choke

United Kingdom

Patty Chondros

Australia

Virasakdi Chongsuvivatwong

Thailand

Imti choonara

United Kingdom

Patricia Chou

United States of America

Shahanaz chowdhury

Bangladesh

Mushtaque Chowdhury

Thailand

Gerardo Chowell

United States of America

Karl Bang Christensen

Denmark

Johnson Christian

Benin

Parul Christian

United States of America

Erik Christiansen

Denmark

Kun-Yang Chuang

Taiwan

Michael Chung

United States of America

Ka Fai Chung

Hong Kong

Bruno Christian Ciancio

Sweden

Theodore Cicero

United States of America

Renata Cifkova

Czech Republic

Manuel Cifuentes

United States of America

Donna Ciliska

Canada

Arzum Ciloglu

United States of America

Maria Grazia Ciufolini

Italy

Jesse Clark

United States of America

Andrew Clark

France

Tainya Clarke

United States of America

Thomas Classen

Germany

Bjorgulf Claussen

Norway

Els Clays

Belgium

Christopher John Clements

Australia

Stacy Clemes

United Kingdom

Susan Clifford

Australia

Jane Clougherty

United States of America

Caroline Cobb

United States of America

Frank Cobelens

Netherlands

Manuel Coelho e Silva

Portugal

Tim Colbourn

United Kingdom

Graham Colditz

United States of America

Andrew Coldman

Canada

Donald Cole

Canada

Brenda Coleman

Canada

Priscilla Coleman

United States of America

Vittoria Colizza

France

Francisco Collazos

Spain

Joanne Colt

United States of America

Erika Comasco

Sweden

Pietro Comba

Italy

John Condon

Australia

James Cone

United States of America

Francesca Conradie

South Africa

Penny Cook

United Kingdom

John Cook

United States of America

Mary Cooke

United Kingdom

Diane Cooper

South Africa

Ray Copes

Canada

Kirsten Coppell

New Zealand

Marine Corbin

France

Kirsten Corder

United Kingdom

Adrijana Corluka

United States of America

Morna Cornell

South Africa

Patrick Corrigan

United States of America

Colleen Corte

United States of America

Andrea A. Cortinois

Canada

Kristen Cosselman

United States of America

Mary-Jean Costello

Canada

Guillaume Coudevylle

Guadeloupe

David Coultas

United States of America

Simon Coulton

United Kingdom

Frances Cowan

United Kingdom

Benjamin Cowling

Hong Kong

Janneke Cox

Netherlands

Angela Craigie

United Kingdom

MeLisa Creamer

United States of America

André Crepaldi

Brazil

Christine Critchley

Australia

Leonard Crocombe

Australia

Ilana Crome

United Kingdom

Wendy Cronin

United States of America

Angela Mary Crook

United Kingdom

Diana Cross

United States of America

Catriona Crossan

United Kingdom

Mario Cruciani

Italy

Rik Crutzen

Netherlands

Fuqiang Cui

China

Stephane Cullati

Switzerland

Michael Cummings

United States of America

Robert Cummins

Australia

David Cundiff

United States of America

Douglas Curran-Everett

United States of America

Marian Currie

Australia

Jeffrey Curtis

United States of America

Brian Custer

United States of America

Francois Dabis

France

Darren Dahly

United Kingdom

Francesco D’Aiuto

United Kingdom

Luigino Dal Maso

Italy

Jean Dallongeville

France

Jeanette Daly

United States of America

Amy Damashek

United States of America

Sarah Damery

United Kingdom

Gianfranco Damiani

Italy

Meredith D’Amore

United States of America

Fortunato D’Ancona

Italy

Barbara Dancy

United States of America

Jeff Dang

United States of America

Shaonong Dang

China

Karen Daniels

South Africa

Zuzana Dankulincova Veselska

Slovakia

Marie- Laure Dardé

France

Murari Das

Nepal

Kaberi Dasgupta

Canada

Nabarun Dasgupta

United States of America

Saeed Dastgiri

Iran

Sangeeta Dave

United Kingdom

Gareth Davey

Hong Kong

Melissa Davey-Rothwell

United States of America

Michael Davidsen

Denmark

Philip Davies

Belgium

Carolyn Day

Australia

Hiram de Almeida Jr

Brazil

Wout de Boer

Switzerland

Angela de Boer

Netherlands

Raquel De Boni

Brazil

Ayesha De Costa

Sweden

Maximilian de Courten

Denmark

Cees de Graaf

Netherlands

Hendrik Dirk de Heer

United States of America

Pieter de Jager

South Africa

Andrew de la Torre

United States Minor Outlying Islands

Dario De Medici

Italy

Augusto Cesar de Moraes

Brazil

Jeroen de Munter

Sweden

Judith de Niet

Canada

Angelique de Rijk

Netherlands

Emely De Vet

Netherlands

Elisabetta de Vito

Italy

Esther de Vries

Netherlands

Gerard de Vries

Netherlands

Chiara de Waure

Italy

Jeroen de Wilde

Netherlands

Astrid de Wind

Netherlands

Leonore M. de Wit

Netherlands

Kyle De Young

United States of America

Wesley Dean

United States of America

Kevin Deane

United States of America

Kora DeBeck

Canada

Jessika Deblonde

Belgium

Michele Decker

United States of America

Johan Decruyenaere

Belgium

Jacob Dee

United States of America

Kathleen Deering

Canada

Benedicte Deforche

Belgium

Louisa Degenhardt

Australia

Olaf Dekkers

Netherlands

Patricia Dekkers-Sanchez

Netherlands

Lilliana Del Busso

Norway

Pierre Delanaye

Belgium

Tom Delbanco

United States of America

Hélène Delisle

Canada

Robert Dellavalle

United States of America

Lotta Dellve

Sweden

Thérèse Delvaux

Benin

Stefaan Demarest

Belgium

Nadia Demarteau

Belgium

Alain Demers

Canada

Vittorio Demicheli

Italy

Andrey Demin

Russian Federation

Saskia Den Boon

Uganda

Elizabeth Denney-Wilson

Australia

Lynette Denny

South Africa

Geoff Der

United Kingdom

Cheryl Der Ananian

United States of America

Kebede Deribe

Ethiopia

Amare Deribew

Ethiopia

Kathryn Derose

United States of America

Olufemi Desalu

Nigeria

Carol DeSantis

United States of America

Jean-Claude Desenclos

France

Yadeta Dessie

Ethiopia

Sarah Isabela Detaille

Netherlands

Roger Detels

United States of America

Natalie DeWitt

United States of America

Aldo Dr Carlo

Italy

Domenico Di Raimondo

Italy

Asuncion Diaz

Spain

Rodrigo Díaz Olmos

Brazil

Fredi Alexander Diaz-Quijano

Colombia

Carlo DiClemente

United States of America

Triantafillos Didangelos

Greece

Patricia Dietz

United States of America

David Diez

United States of America

Peter John Diggle

United Kingdom

Cyril Dim

Norway

Charles DiMaggio

United States of America

Carolyn Dimitri

United States of America

Maria de Fátima Diniz

Brazil

Rylee Dionigi

Australia

Helen Dixon

Australia

Bosiljka Djikanovic

Serbia

Olgica Djurkovic-Djakovic

Serbia

Khoi Do

Australia

Catherine Dodds

United Kingdom

Kirsten Doherty

Ireland

Kate Dolan

Australia

Sheila Dollard

United States of America

David Dolling

United Kingdom

Stephan Dombrowski

United Kingdom

Àngela Domínguez

Spain

Carolin Donath

Germany

Nathalie Donders

Netherlands

Brent Donnellan

United States of America

Leeann Donnelly

Canada

Tam Truong Donnelly

Canada

Martin Donoghoe

Denmark

Danny Dorling

United Kingdom

Diana Dorstyn

Australia

Peter Doshi

United States of America

Chyke Doubeni

United States of America

Jacqueline Doumit

Lebanon

Evangelia Dounousi

Greece

Terence Dovey

United Kingdom

William Dow

United States of America

Scott Dowell

United States of America

Soo Downe

United Kingdom

Amy Downing

United Kingdom

Jennifer Downing

United Kingdom

Therese Dowswell

United Kingdom

Pat Doyle

United Kingdom

Nico Dragano

Germany

Mitra Drakulovic

Serbia

Beverly Draper

South Africa

Catherine Draper

South Africa

Jessica Draughon

United States of America

Robert Dreibelbis

United States of America

Eva Drevenhorn

Sweden

Julian Drewe

United Kingdom

Steven Drews

Canada

Tim Driscoll

Australia

David Dror

India

Ernest Drucker

United States of America

Marjan Drukker

Netherlands

Lisanne du Plessis

South Africa

Ana Duarte

United Kingdom

Eve Dubé

Canada

Ann-Sofi Duberg

Sweden

Dean Dudley

Australia

Samuel Dumith

Brazil

Michael Duncan

United Kingdom

Scott Duncan

New Zealand

Mitch J Duncan

Australia

Dustin Duncan

United States of America

Tinashe Dune

Australia

David Dunger

United Kingdom

Esther Dungumaro

Tanzania

Casey Durand

United States of America

Michael Dworsky

United States of America

Debra Dwyer

United States of America

David Dzewaltowski

United States of America

Lisa Eaton

United States of America

Kenneth Eaton

United Kingdom

Shahul Ebrahim

United States of America

Martin Eccles

United Kingdom

Justin Basile Echouffo-Tcheugui

United States of America

Alan Eckeli

Brazil

Michael Eddleston

United Kingdom

Mary Edginton

South Africa

Wijnand Eduard

Norway

Duncan Edwards

United Kingdom

Brenda K Edwards

United States of America

Phil Edwards

United Kingdom

Matt Egan

United Kingdom

Grace Egeland

Canada

Torbjørn Moe Eggebø

Norway

Garry Egger

Australia

Jason Egginton

United States of America

Ulrike Ehlert

Switzerland

Maria Ekstrand

United States of America

Mazen El Ghaziri

United States of America

Karima El Rhazi

Morocco

Susan Elden

United Kingdom

Frank Elgar

Canada

Bernice Elger

Switzerland

Carol El-Hayek

Australia

Johannes Elias

Germany

Michele Eliason

United States of America

Mats Eliasson

Sweden

Brett Ellis

United States of America

Lis Ellison-Loschmann

New Zealand

Aiman El-Saed

Saudi Arabia

Abdulrahman El-Sayed

United States of America

Manal El-Sayed

Egypt

Stacy Eltiti

United States of America

Tara Elton-Marshall

Canada

Rune Elvik

Norway

Tim Elwell-Sutton

Hong Kong

Paul Emerson

United States of America

Jerome Endrass

Switzerland

Bo Engdahl

Norway

Ola Engelsen

Norway

Elizabeth England

United Kingdom

Patrice Engle

United States of America

Peter English

United Kingdom

Rachel Eni

Canada

Bircan Erbas

Australia

Cihangir Erem

Turkey

Charli Eriksson

Sweden

Malin Eriksson

Sweden

Tadese Asfaw Erku

Ethiopia

Annette Erlangsen

Denmark

Sebhat Erqou

United States of America

Nicole Errett

United States of America

Guido Erreygers

Belgium

Jan Jaap H.M. Erwich

Netherlands

Javier I Escobar

United States of America

Brigitte Escoubet

France

Olapeju Esimai

Nigeria

Laura Esmail

United States of America

Susanna Esposito

Italy

Birgitta Essén

Sweden

Marie-Louise Essink-Bot

Netherlands

Agustín Estrada-Peña

Spain

Paul Etkind

United States of America

Meirion Evans

United Kingdom

Catrin Evans

United Kingdom

Juliet Evans

South Africa

Elizabeth Evans

United Kingdom

Charlotte Evans

United Kingdom

Sara Evans-Lacko

United Kingdom

Frank Eves

United Kingdom

Daniel Exeter

New Zealand

Amy Eyler

United States of America

Helen Eyles

New Zealand

Nadine Ezard

Australia

Lars Thore Fadnes

Norway

Fabrizio Faggiano

Italy

Elham Faghihimani

Iran

Jamshid Faghri

Iran

Tala Fakhouri

United States of America

Lavinia Falese

Italy

Freddy Falez

Belgium

Li-Qun Fang

China

Ya Fang

China

Allan G Farman

United States of America

Zafar Fatmi

Pakistan

Adesegun Fatusi

Nigeria

Daniel Faurholt-Jepsen

Denmark

Olufunmilayo Fawole

Nigeria

Michael Fay

United States of America

Seena Fazel

United Kingdom

Awat Feizi

Iran

Marilyn Felkner

United States of America

Mogens Fenger

Denmark

Trevor Ferguson

Jamaica

David Fergusson

New Zealand

Frans J.M. Feron

Netherlands

Sandra Roberta Gouvea Ferreira

Brazil

Efigênia Ferreira

Brazil

Alejandro Ferrer San Juan

Spain

Cleusa Ferri

United Kingdom

Jane Ferrie

United Kingdom

Jason Ferris

Australia

Karin Festin

Sweden

Netsanet Fetene

Ethiopia

Paul Alozie Feyi-Waboso

Nigeria

Sarah Fidler

United Kingdom

Stephen Field

Canada

Robert Field

United States of America

James Fielding

Australia

Barbara Fiese

United States of America

Ulrike Fillinger

United Kingdom

Julia Finkelstein

United States of America

Andrea Finlay

United States of America

Sarah Finocchario Kessler

United States of America

Jordan Firestone

United States of America

Christa Fischer Walker

United States of America

Helen Fisher

United Kingdom

Colleen Fisher

United States of America

Susan Fisher-Hoch

United States of America

Patricia Fitzpatrick

Ireland

Sarah Flanagan

United Kingdom

William Flanders

United States of America

Steffen Flessa

Germany

Olivia Fletcher

United Kingdom

Nils Fleten

Norway

Jennifer Flood

United States of America

Matilda Florentin

Greece

Stephen Flores

United States of America

Alex Florindo

Brazil

George Fodor

Canada

Andrew Fogarty

United Kingdom

Andrea Foli

Italy

Francesca Foltran

Italy

Kevin Fontaine

United States of America

Kelsie Forbush

United States of America

Nathan Ford

South Africa

John Foreyt

United States of America

Maren Formazin

Germany

Alice Forster

United Kingdom

Paola Forti

Italy

Emma Foster

United Kingdom

Deshka Foster

United States of America

Charlie Foster

United Kingdom

Jean-Christophe Fotso

Kenya

Kathleen Fox

United States of America

Pieter Fraaij

Netherlands

Renerio Fraguas

Brazil

Philippe Fraisse

France

Elisabeth França

Brazil

Elisabetta Franco

Italy

Manuel Franco

Spain

Richard Franklin

Australia

Eelco Franz

Netherlands

John Fraser

Australia

Paulo Frazao

Brazil

Rosanne Freak-Poli

Australia

Becky Freeman

Australia

Teresa Freire

Portugal

Maria do Carmo Freire

Brazil

Clare French

United Kingdom

Davina French

Australia

Atle Fretheim

Norway

Morten Frisch

Denmark

Sari Fröjd

Finland

Marcela Fu

Spain

Chaowei Fu

China

Adrian Fuente

Australia

Hans-Martin Fuessel

Denmark

Jong-Ling Fuh

Taiwan

Yoshiharu Fukuda

Japan

Kathleen Fullerton

United States of America

Thomas Fungwe

United States of America

Claire Furlong

United Kingdom

Jon Furuno

United States of America

Lance Gable

United States of America

Suresh Gadde

United States of America

Anne Gadermann

Canada

Sylvie Gadeyne

Belgium

Giovanni Gaeta

Italy

Anita Gagnon

Canada

Omar Galarraga

United States of America

Carol Galletly

Vanuatu

Karine Gallopel-Morvan

France

Silvano Gallus

Italy

Vijay Ganji

United States of America

Marie Gantz

United States of America

Yan Gao

China

Nuria Garatachea

Spain

Jesus M Garcia Calleja

Switzerland

Maria Luiza Garcia Rosa

Brazil

Luis García-Olmos

Spain

Maria Paz Garcia-Portilla Gonzalez

Spain

Phillip Gardiner

United States of America

Sara Garfield

United Kingdom

Genevieve Gariepy

Canada

Gulcan Garip

United Kingdom

Andy Garman

United States of America

Tania Gaspar

Portugal

Joseph Gasper

United States of America

Eleanor Gaunt

United Kingdom

Vilma Gawryszewski

United States of America

Anelise Gaya

Brazil

Thomas Gaziano

United States of America

Andrea Gazzinelli

Brazil

Klaus Gebel

Australia

Rolf Gedeborg

Sweden

paul wenzel geissler

United Kingdom

Becky Genberg

United States of America

Joan Gene Badia

Spain

Clemon George

Canada

Elizabeth George

United Kingdom

Sanne Gerards

Netherlands

Markus Gerber

Switzerland

Linda Gerber

United States of America

Danielle German

United States of America

Dorota Gertig

Australia

Francesco Gesualdo

Italy

Neil Gesundheit

United States of America

Yigzaw Kebede Gete

Ethiopia

Peter Gething

United Kingdom

Elena Ghanotakis

United States of America

Hadi Ghasemi

Iran

Rohini Ghosh

India

Sanjib Ghosh

India

Sharon Ghuman

United States of America

Luana Giatti

Brazil

Pedro Giavina-Bianchi

Brazil

Bryan Gibson

United States of America

Faith Gibson

United Kingdom

Jasmine Gideon

United Kingdom

Christopher Gidlow

United Kingdom

Andreas Gies

Germany

Norman Giesbrecht

Canada

Hazel Gilbert

United Kingdom

Mahée Gilbert-Ouimet

Canada

Alfredo Gilio

Brazil

Melissa Gilkey

United States of America

Tim Gill

Australia

Tiffany Gill

Australia

Melissa Gilliam

United States of America

Conor Gilligan

Australia

Robert Gilman

United States of America

Pedro Gil-Monte

Spain

Lucy Gilson

South Africa

Suely Godoy Agostinho Gimeno

Brazil

Rosa Gini

Italy

Gabriele giorgi

Italy

Enrico Girardi

Italy

Alan Girling

United Kingdom

Samuel Girma

Ethiopia

Gunnar Gislason

Denmark

Marina Giuliano

Italy

Peter Glasauer

Italy

Danya Glaser

United Kingdom

Nancy Glass

United States of America

Nick Glozier

Australia

Joseph Gnanaraj

United States of America

Roberto Gnavi

Italy

Wendy Gnich

United Kingdom

Fabriziomaria Gobba

Italy

Tesfaye Gobena

Ethiopia

Rosa Gofin

United States of America

Patricia Goggin

Canada

Deborah T Gold

United States of America

Rajna Golubic

United Kingdom

Mariana Golumbeanu

Romania

Fabio Gomes

Brazil

Scarlett Gomez

United States of America

Javier Gomez de Terreros

Spain

Maciej Goniewicz

Poland

Anna Good

United Kingdom

Ana Goode

Australia

Robert Goodman

United States of America

Patrick Goodman

Ireland

Catherine Goodman

Kenya

Daniel Goon

South Africa

Pierre Goovaerts

United States of America

Trish Gorely

United Kingdom

Anna Gorter

Netherlands

Mercè Gotsens

Spain

Hebe Gouda

Australia

Alessandra Goulart

Brazil

Alessandra Goulart

Brazil

Fares Gouzi

France

Jeff Gow

South Africa

Agnes Gozdzik

Canada

Kristina Grabbe

United States of America

Luigi Gradoni

Italy

Silke Graeser

Germany

Samantha Graff

United States of America

Mariaelisa Graff

United States of America

Petra Graham

Australia

Michael Grandner

United States of America

William B. Grant

United States of America

Carol Grant-Pearce

United Kingdom

Jane Grassley

United States of America

Damion Grasso

United States of America

Cindy Gray

United Kingdom

Annmarie Grealish

United Kingdom

Colin Greaves

United Kingdom

Michael James Green

United Kingdom

Jennifer Green

United States of America

John Greenaway

United Kingdom

Ted Greiner

Korea South

Christine Grella

United States of America

Pippa Grenfell

United Kingdom

Karen Grepin

United States of America

Jacoba P Greving

Netherlands

Gloria Grice

United States of America

Jessica Grieger

Australia

Jeffrey Grierson

Australia

Pippa Griew

United Kingdom

Sarah Griffin

United States of America

James Griffith

United States of America

Ulla Griffiths

United Kingdom

sian Griffiths

Hong Kong

Diana Grigsby-Toussaint

United States of America

Martin Peter Grobusch

Netherlands

Iris Groeneveld

Netherlands

Peter Groenewegen

Netherlands

Rolf Groenwold

Netherlands

Danielle Groffen

Netherlands

Wolfgang Gruber

Germany

Yian Gu

United States of America

Danan Gu

United States of America

Secondo Guaschino

Italy

Ricardo Guerra

Brazil

Richard Guerrant

United States of America

Fernando Guerrero-Romero

Mexico

Idris Guessous

Switzerland

Lin Guey

United States of America

Martin Guhn

Canada

Ashleigh Guillaumier

Australia

Gabriel Gulis

Denmark

Freedom Nkhululeko Gumedze

South Africa

David Gunnell

United Kingdom

How-Ran Guo

Taiwan

Yi Guo

China

Fangjian Guo

United States of America

Rajeev Gupta

India

Ulvi Gursoy

Finland

Anup Gurung

Papua New Guinea

Gustavo Gusso

Brazil

Per E Gustafsson

Sweden

Jillian (Jill) Guthrie

Australia

Bruce Guthrie

United Kingdom

Mariana Gutierres Arteiro da Paz

Brazil

Antonio Gutierrez

United States of America

Rebecca Guy

Australia

Cyril Guyard

Canada

Emma Guymer

Australia

Stella Gwini

Australia

Elizabeth Gyllstrom

United States of America

Flora Haaijer-Ruskamp

Netherlands

Sandra Hacon

Brazil

Ella Haddad

United States of America

Andrew Haddow

United States of America

George Haddow

United States of America

James Hadler

United States of America

Craig Hadley

United States of America

Gareth Hagger-Johnson

United Kingdom

Maria Hagstromer

Sweden

Myung-Il Hahm

Korea South

Robert Hahn

United States of America

Majid Hajifaraji

Iran

Amal Krishna

**Halder** Bangladesh

Kai Haldre

Estonia

Jason Halford

United Kingdom

Alan Hall

United States of America

Kevin Hall

United States of America

H Irene Hall

United States of America

Dagmar Haller

Switzerland

Katherine Halliday

United Kingdom

Jaana Halonen

Finland

Jena Hamadani

Bangladesh

Nobuyuki Hamajima

Japan

Ahmad Hamdy

Egypt

Anne Hammarström

Sweden

Sung Nim Han

Korea South

Amresh Hanchate

United States of America

Trevor Hancock

Canada

Tonelle Handley

Australia

Ivan Hanigan

Australia

David Hanley

Canada

Phil Hanlon

United Kingdom

Philip Hannaford

United Kingdom

Colleen Hanrahan

United States of America

Claus D. Hansen

Denmark

Anders Hansen

United Kingdom

Nathan Hansen

United States of America

Zaeem Haq

United States of America

Tetsuo Harada

Japan

Sarah Hardcastle

United Kingdom

Katherine Hardcastle

United Kingdom

Karen Hardee

United States of America

Anita Hardon

Netherlands

Louise Hardy

Australia

Sam Harper

Canada

Jane Harries

South Africa

Kathleen Harrington

United States of America

Margaret Harris

Switzerland

Jennifer Harris

United States of America

Magdalena Harris

United Kingdom

Ingunn Harstad

Norway

Jaime Hart

United States of America

Rosane Harter Griep

Brazil

William Hartmann

United States of America

Samuel Harvey

United Kingdom

Jean Harvey-Berino

United States of America

Amy Jo Harzke

United States of America

Ahmed Hashim

Canada

Hideki Hashimoto

Japan

Ali Hashtroufi

United Kingdom

Abul Hasnat

Bangladesh

Per Erlend Hasvold

Norway

Angelos Hatzakis

Greece

Jari Haukka

Finland

Alys Havard

Australia

Anna Hawkes

Australia

Gail Hawkes

Australia

Graeme Hawthorne

Australia

Phillipa Hay

Australia

Abby Haynes

Australia

Ping He

China

Na He

China

Meizi He

United States of America

Genevieve Healy

Australia

Todd Heard

Australia

Gregory Heath

United States of America

Matthew Heaton

United States of America

Kathy Hebert

United States of America

Ulrich Hegerl

Germany

Behzad Heidari

Iran

Wieke H. Heideman

Netherlands

Christin Heidemann

Germany

Robert Heimer

United States of America

Katie Heinrich

United States of America

Charreire Hélène

France

Asgeir R. Helgason

Sweden

Kevin Helgeson

United States of America

Wiebke Hellenbrand

Germany

Stefanie Helmer

Germany

Max Henderson

United Kingdom

Pascal Hendrikx

France

Lisa Henriksen

United States of America

Emilie Henry

France

Niel Hens

Belgium

Jane Herlihy

United Kingdom

Todd Herrenkohl

United States of America

Rachel Herring

United Kingdom

Patti Herring

United States of America

Tiberiu Hershcovici

United States of America

Jean Hertzberg

United States of America

Sonja Hess

United States of America

Morten Hesse

Denmark

Paul Hewitson

United Kingdom

Peter Heywood

Australia

Judith Hibbard

United States of America

Marieke Hiemstra

Netherlands

Roger Hilfiker

Switzerland

James Hill

United States of America

Zelee Hill

United Kingdom

Frances Hillier

United Kingdom

Amy Hillier

United States of America

Melvyn Hillsdon

United Kingdom

David Himmelgreen

United States of America

Daniel Hind

United Kingdom

Trina Hinkley

Australia

Valeria Hirschler

Argentina

Yael Hirsch-Moverman

United States of America

Mirja Hirvensalo

Finland

Harriet Hiscock

Australia

Eva Hnizdo

United States of America

Samuel Ho

United States of America

Erin Hobin

Canada

Allison Hodge

Australia

Michael Hodgson

United States of America

Victor Hoe

Malaysia

Christian Hoebe

Netherlands

Kristin Hoeft

United States of America

Janet Hoek

New Zealand

Daniel Hoffman

United States of America

Geoffrey Hoffman

United States of America

Kate Hoffman

United States of America

Caisa Hofgren

Sweden

Le Van Hoi

Viet Nam

Michael Holick

United States of America

Paula Holland

United Kingdom

Samantha Hollingworth

Australia

Gerd Holmboe-Ottesen

Norway

Jostein Holmen

Norway

John Holmes

United Kingdom

Kristina Holmgren

Sweden

Martin Holzhausen

Germany

Caroline Homer

Australia

Greg Homish

United States of America

Yuling Hong

United States of America

Tollula Honi

South Africa

Heidi Hopkins

Uganda

Monjurul Hoque

South Africa

Yoko Hori

Japan

Rebecca Horn

United Kingdom

John Hornberger

United States of America

Maria Horne

United Kingdom

Jason Horsley

United Kingdom

John Hoskins

United Kingdom

Nazir Hossain

Canada

Farhad Hosseinpanah

Iran

Mohammad Javad Hosseinzadeh

Iran

Jonathan Houdmont

United Kingdom

Inge Houkes

Netherlands

Janie Houle

Canada

Ciska Hoving

Netherlands

Bethany Howard

Australia

Annabel Howard

Netherlands

Philippa Howden-Chapman

New Zealand

Edmund Howe

United States of America

Laura Howe

United Kingdom

Bing-Mu Hsu

Taiwan

Guoqing Hu

China

Zhiyong Hu

United States of America

Min-Feng Huang

Taiwan

Yueqin Huang

China

Song Lih Huang

Taiwan

Christer Hublin

Finland

Fatma Huffman

United States of America

Karen Hughes

United Kingdom

Caitlin Hughes

Australia

Nathalie Huguet

United States of America

Martijn Huisman

Netherlands

Claire Hulme

United Kingdom

Arun Humne

India

Hilton Humphries

South Africa

Debbie Humphries

United States of America

Vanora Hundley

United Kingdom

Hien-Ching Hung

Taiwan

Sally Hunter

Australia

Jennifer Hunter

Australia

Ruth Hunter

United Kingdom

Rafat Hussain

Australia

Amanda Hutchinson

Australia

Dieu Huynh

Viet Nam

Sel Hwahng

United States of America

Terri Hyde

United States of America

Hyacinth Ichoku

Nigeria

Christopher William Idemudia

United Kingdom

Erhabor Idemudia

South Africa

Martin Iguchi

United States of America

Naoki Ikegami

Japan

Aamer Imdad

United States of America

Marie-Gloriose Ingabire

Canada

Maria Ingemarson

Sweden

Nadia Inglis

United Kingdom

Akiomi Inoue

Japan

Shigeru Inoue

Japan

Hazel Inskip

United Kingdom

Giovanni Iolascon

Italy

Giuseppe Ippolito

Italy

Masahiro Irie

Japan

Jennifer D. Irwin

Canada

Kevin Irwin

United States of America

Warnow Elon Isaac

Nigeria

Petros Isaakidis

India

Deus Ishengoma

Tanzania

John Iskander

United States of America

Md Shahidul Islam

Australia

Abdul Rashid Ismail

Malaysia

Leyla Ismayilova

United States of America

Saadou Issifou

Gabon

Claire Italiano

Australia

Yuri Ito

Japan

Motoki Iwasaki

Japan

Maria Izar

Brazil

Kazuhiro Izawa

Japan

Alberto Izzotti

Italy

Paulo de Andrade Jacinto

Brazil

Suzanne Jackson

Canada

Cristina Jacob

Brazil

Tazeen Jafar

Pakistan

Jagnoor Jagnoor

Australia

Russell Jago

United Kingdom

Albrecht Jahn

Germany

Irena Jakopanec

Norway

Paul James

Canada

Amy James

United States of America

KS James

India

Monika Janda

Australia

Soong-nang Jang

Korea South

Urban Janlert

Sweden

Ian Janssen

Canada

Edward Janus

Bahamas

Edward Janus

Australia

Antoine Jaquet

France

Paulo Cesar Jardim

Brazil

Brant Jarrett

United States of America

Somchit Jaruratanasirikul

Thailand

Nathalie Jauvin

Canada

Radhakrishnan Jayakrishnan

India

Upali W. Jayasinghe

Australia

Ranil Jayawardena

Sri Lanka

Oluwemimo Jayeoba

Canada

Steve Jefferys

United Kingdom

Michael Jelinek

Australia

Tim Jelleyman

New Zealand

Emily Jenkins

Canada

Rachel Jenkins

United Kingdom

Samuel Jenness

United States of America

Chris Jensen

Denmark

Jorgen Dejgaard Jensen

Denmark

Jan L Jensen

Canada

Anne Karen Jenum

Norway

David Jernigan

United States of America

Gerald Jerome

United States of America

Weiping Jia

China

zhongwei jia

China

Joanna Jiang

United States of America

Shiwen Jiang

China

Stephanie Jilcott Pitts

United States of America

Rodrigo Jimenez Garcia

Spain

David Jiménez-Pavón

Spain

Fengyi Jin

Australia

Ruxana Jina

South Africa

Kitti Jirarattanaphochai

Thailand

Mark Jit

United Kingdom

Moyez Jiwa

Australia

Elise Johansen

Switzerland

Oystein Haarklau Johansen

Norway

Pia Johansson

Sweden

Gun Johansson

Sweden

Garry John

United Kingdom

Ulrich John

Germany

Roar Johnsen

Norway

Ann-Christin Johnson

Sweden

Timothy Johnson

United States of America

Karen Johnson

United States of America

Aaron Johnson

United States of America

Joy Johnson

Canada

Rory Johnston

Canada

Vanessa Johnston

Australia

Isabelle Joing

France

Markus Jokela

Finland

Brad Jokisch

United States of America

Damien Jolley

Australia

Bruce Jonas

United States of America

Heidi Jones

United States of America

Martyn Jones

United Kingdom

Lisa Jones

United Kingdom

Cornelia Jonker

South Africa

Anna Jönsson

Sweden

Rachel Jordan

United Kingdom

Michael Jordan

China

Marie Birk Jorgensen

Denmark

Malin Josephson

Sweden

Kaumudi Joshipura

United States of America

Eric Jougla

France

Tanisha Jowsey

Australia

Ali Judd

United Kingdom

Knud Juel

Denmark

Claudia Julio

Portugal

Dolors Juvinya

Spain

Zubair Kabir

Ireland

Zubair Kabir

Ireland

Andrew. T Kaczynski

United States of America

Umesh Kadam

United Kingdom

Anthony Kafatos

Greece

Soo KahLeng

Malaysia

Bernard Kaic

Croatia

Sylvia Kairouz

Canada

Rose Kakai

Kenya

Lisa Kakinami

Canada

László Kalabay

Hungary

Eleni Kalamara

Portugal

John Kalbfleisch

United States of America

Dorota Kaleta

Poland

Linda Kaljee

United States of America

Gurvinder Kalra

India

Ofra Kalter - Leibovici

Israel

Sebastiana Kalula

South Africa

Masamitsu Kamada

Japan

Shaden Kamhawi

United States of America

Srinivasan Kannan

India

Thomas Kantermann

Germany

Suman Kanungo

India

Uraiwan Kanungsukkasem

Thailand

Alan Kaplan

Canada

Bulent Karadag

Turkey

Asterios karagiannis

Greece

Majid Karandish

Iran

Zunaid Karar

Bangladesh

Mojgan Karbakhsh

Iran

Mojgan Karimi zarchi

Iran

Saffron Karlsen

United Kingdom

Björn Karlson

Sweden

Bernt Karlsson

Sweden

Jordan Karp

United States of America

Susan Kasedde

United States of America

Nina Kaseva

Finland

Taha Kass-Hout

United States of America

Alexander Katalinic

Germany

Kota Katanoda

Japan

Jun Kato

Japan

Jodie Katon

United States of America

Niki Katsiki

Greece

Ioanna Katsounari

Cyprus

Alan Katz

United States of America

Judith Katzenellenbogen

Australia

Laura Kauhanen

Finland

Prabhdeep Kaur

India

Method Kazaura

Tanzania

Lawrence Ndekeleni Kazembe

South Africa

Wojciech Kazimierz

Poland

Michael Keall

New Zealand

Catherine Keating

Australia

Michelle Kegler

United States of America

Helen Keleher

Australia

Bridget Kelly

Australia

Heath Kelly

Australia

Joel Kelso

Australia

Anna Kemp

Australia

Han CG Kemper

Netherlands

Hal Kendig

Australia

Andre Pascal Kengne

South Africa

Ryan David Kennedy

United States of America

Ruth Keogh

United Kingdom

Kate Kerber

South Africa

Michelle Kermode

Australia

Jacqueline Kerr

United States of America

Kiarri Kershaw

United States of America

Ronald Kessler

United States of America

Katherine Keyes

United States of America

Zarnie Khadjesari

United Kingdom

Samoel Khamadi

Kenya

Amina Khambalia

Australia

Asad Khan

Australia

Mohammad Khan

Korea South

Mobarak Khan

Germany

Salwa Khan

United States of America

Masuma Akter Khanam

Bangladesh

Ayesha BM Kharsany

South Africa

Davoud Khorasani-Zavareh

Sweden

Babak Khoshnood

France

Sadik Khuder

United States of America

James Kiarie

Kenya

Michele Kiely

United States of America

Masahiro Kihara

Japan

Molly Kile

United States of America

Young-Min Kim

Korea South

Daniel Kim

United States of America

Jinhyun Kim

United States of America

Andrea Kim

United States of America

Jin Hee Kim

Korea South

Jo Kimber

Australia

April Kimmel

United States of America

Joyce Kinaro

Kenya

Terry Kind

United States of America

Bill King

Australia

Malcolm King

Canada

Ann Louise Kinmonth

United Kingdom

Patrick Kinney

United States of America

Tarja I Kinnunen

Finland

Eugene Kinyanda

Uganda

Susan Kippax

Australia

Michael Kibet Kiptoo

Kenya

Pia Kivelä

Finland

Tamar Klaiman

United States of America

Henrikje Klasen

Netherlands

Jeffrey Klausner

United States of America

Nanne Kleefstra

Netherlands

Michael Klein

Canada

Elizabeth Klein

United States of America

Christina Kleiser

Germany

Zalika Klemenc-Ketis

Slovenia

Bart Klijs

Netherlands

Kerstin Klipstein-Grobusch

South Africa

Marjan Kljakovic

Australia

Marit Knapstad

Norway

Lee Knifton

United Kingdom

Stephen Knight

South Africa

Julia Knight

Canada

Kai Knudsen

Sweden

Ying-Chin Ko

Taiwan

Daiki Kobayashi

Japan

Sarah Kobrin

United States of America

Vera Koch

Brazil

Mihail Kochubovski

Macedonia

Robin Köck

Germany

Paul Kocken

Netherlands

Jillian Clare Kohler

Canada

Heli Koivumaa-Honkanen

Finland

Takehiko Koji

Japan

Constantinos Kokkinos

Greece

Emilia Kolarzyk

Poland

Tracy Kolbe-Alexander

South Africa

Elin Kolle

Norway

Alan Kolok

United States of America

Dimitrios Komilis

Greece

Ina Koning

Netherlands

Ayumi Kono

Japan

Flemming Konradsen

Denmark

Malcolm Koo

Canada

Xander Koolman

Netherlands

Hannu Koponen

Finland

Sjaan Koppel

Australia

Rachael Korcha

United States of America

Katarzyna Kordas

United States of America

Michael Kortt

Australia

Kanyarat Korwanich

Thailand

Karolina Kósa

Hungary

Michael Kostapanos

Greece

Konstantinos Kostikas

Greece

Tomasz Kostka

Poland

Lidia Kostyniuk

United States of America

Anita Kothari

Canada

Vasilios Kotsis

Greece

Thomas Kottke

United States of America

Marinda Kotze

South Africa

Efstathios Koulouridis

Greece

John Koval

Canada

Augustine Kposowa

United States of America

Douglas Krakower

United States of America

Alex Kral

United States of America

Katharina Kranzer

United Kingdom

Allan Krasnik

Denmark

Amy Krentzman

United States of America

Anand Krishnan

India

Andrea Kriska

United States of America

Ivar Sonbo Kristiansen

Norway

Alfgeir Logi Kristjansson

United States of America

Rungroj Krittayaphong

Thailand

Willemieke Kroeze

Netherlands

Thilo Kroll

United Kingdom

Erkki Kronholm

Finland

Jeroen Kroon

Australia

Janet Krska

United Kingdom

Margaret Elizabeth Kruk

United States of America

Edward Krupat

United States of America

Gina Kruse

United States of America

Kazumi Kubota

Japan

Elizabeth Kuhl

United States of America

Louise Kuhn

United States of America

Joe Kuhns

United States of America

Paul Kuijer

Netherlands

Pim Kuipers

Australia

Rajan Kulkarni

United States of America

Hemant Kulkarni

United States of America

Manoj Kumar

Canada

Rajesh Kumar

India

Mahendra Kumar

United States of America

Seema Kumar

United States of America

Abera Kumie

Ethiopia

Ernesta Kunneke

South Africa

Anton Kunst

Netherlands

Peter Kunstadter

Thailand

Chian-Jue Kuo

Taiwan

Varant Kupelian

United States of America

Nyaradzai Edith Kurewa

Zimbabwe

Terhi Kurko

Finland

Craig Kuziemsky

Canada

Kirsti Kvaløy

Norway

Siv Kvernmo

Norway

Lydia Kwak

Sweden

Enid Wai-yung Kwong

Hong Kong

Giuseppe La Torre

Italy

David Laaksonen

Finland

Tiina Laatikainen

Finland

Philippe Lacherez

Australia

Anton Carl Jonas Lager

Sweden

Leizel Lagrada

Philippines

Raija Lähdesmäki

Finland

Eero Lahelma

Finland

Petra H Lahmann

Australia

Dejian Lai

United States of America

Sharon Laing

United States of America

iain lake

United Kingdom

Jeroen Lakerveld

Netherlands

Rajalakshmi Lakshman

United Kingdom

Tai Pong Lam

Hong Kong

Cindy Lam

Hong Kong

Bodil Landstad

Sweden

Marian Lane

United States of America

Ellen Melbye Langballe

Norway

Eva Langeland

Norway

Mike Ralf Langenbach

Germany

Ricky Langley

United States of America

Ann Laramee

United States of America

Linda Larcombe

Canada

Sarah Larney

Australia

Regina LaRocque

United States of America

Amparo Larrauri

Spain

David Larsen

United States of America

Heidi Larson

United Kingdom

Bruce Larson

United States of America

Ann Larson

Australia

Charles Larson

Canada

Matz Larsson

Sweden

Sofie Gillesberg Lassen

Germany

Cia-Hin Lau

Malaysia

Murray Laugesen

New Zealand

Robert Launois

France

Patrizia Laurenti

Italy

Martin Lavallière

Canada

Angela Lawless

Australia

Sharon Lawn

Australia

Taiwo Lawoyin

Nigeria

David Lawrence

Australia

Mark Lawrence

Australia

Lovett Lawson

Nigeria

Chrystalleni Lazarou

Greece

Eduardo Lazcano-Ponce

Mexico

Le Minh Giang

Viet Nam

William Leaf

United States of America

Annette Leclerc

France

Suzanne Leclerc-Madlala

United States of America

Jie-Min Lee

Taiwan

Duk-Hee Lee

Korea South

Kiyoung Lee

Korea South

Yunhwan Lee

Korea South

Byung-Kook Lee

Korea South

Peter Lee

United Kingdom

Paul Lee

Hong Kong

Albert Lee

Hong Kong

Yong-Jae Lee

Korea South

Tony S.H. Lee

Taiwan

Shelley Lees

United Kingdom

Rafael Lefkowitz

United States of America

James Leigh

Australia

Cédric Lemogne

France

Erika Lencova

Czech Republic

Michela Lenzi

Italy

Geraldine Leong

United Kingdom

Michalis Leotsinidis

Greece

Anja Leppin

Denmark

Lori Lerner

United States of America

France Lert

France

Wilma Leslie

United Kingdom

William D. Leslie

Canada

Richard Lester

Canada

Ting Fan Leung

China

Ting Fan Leung

Hong Kong

Amy Leval

Sweden

Tobe Levin

Germany

David Levy

United States of America

Steven Levy

United States of America

R. Jeffrey Lewis

United States of America

Joel Lexchin

Canada

Els Leye

Belgium

Liming Li

China

Zhihua Li

China

Li-ping Li

China

Yanping Li

United States of America

Leah Li

United Kingdom

Nanfang Li

China

Duo Li

China

Jialiang Li

Singapore

Hang Li

China

Shu Qin Li

Australia

Song Liang

United States of America

Wee Liang En Ian

Singapore

Evangelos Liberopoulos

Greece

Ulrik Lidwall

Sweden

Toni Liechty

Canada

Nanna Lien

Norway

Lars Lien

Norway

Angela Liese

United States of America

Gösta Liljeqvist

Australia

Jennifer NW Lim

United Kingdom

Yvonne Ai Lian Lim

Malaysia

Lynette L-Y Lim

Australia

Aldo Lima

Brazil

Rupali Limaye

United States of America

Wen-Yuan Lin

Taiwan

Hua Liang Lin

China

Yusen Lin

Taiwan

Vivian Lin

Australia

Tin-chi Lin

United States of America

Chia-Hsien Lin

Taiwan

Ulf Lindblad

Sweden

Jutta Lindert

Germany

Per Lindqvist

Sweden

Geoff Lindsay

United Kingdom

William Lindsley

United States of America

Jaana Lindström

Finland

Raghu Lingam

United Kingdom

Wendy Lipworth

Australia

Bert Little

United States of America

Xiaodong Liu

China

Ailing Liu

China

Yinliang Liu

China

Hua Liu

United States of America

Jianping Liu

China

Youcheng Liu

United States of America

Jian Liu

Canada

Ruiling Liu

United States of America

Shing-Hwa Liu

Taiwan

Katherine Cherry Liu

Zambia

Liegang Liu

China

Qin Liu

China

Jianghong Liu

United States of America

Alon Livny

Israel

Lisa Lix

Canada

Rickard Ljung

Sweden

Per Ljungman

Sweden

Mathias Loch

Brazil

Sara Lodi

Spain

Hugues Loiseau

France

Yoon Kong Loke

United Kingdom

Getahun Ersino Lombamo

Canada

Carl Lombard

South Africa

Knut Lönnroth

Switzerland

Mike Loosemore

United Kingdom

Pier Luigi Lopalco

Sweden

Joao Lopes

Portugal

Anna Lena Lopez

Philippines

Maria Jose Lopez

Spain

Julio Lopez-Bastida

Spain

Nicolas Lorgnier

United States of America

Dora Loria

Argentina

Olabisi Loto

Nigeria

Francisco Lotufo Neto

Brazil

Wolff Loup

France

Nuno Loureiro

Portugal

Mona Loutfy

Canada

Julia Louw

South Africa

Marian

Loveday

South Africa

Helen Lowey

United Kingdom

Fan Lu

China

Liang Lu

China

David Lubans

Australia

Tom Luben

United States of America

Patricia Lucas

United Kingdom

Daniele Luce

France

Cynthia Lucero

United States of America

John Ludbrook

Australia

Vivian Luft

Brazil

Alessandra Lugo

Italy

Karoline Lukaschek

Germany

Julie Lumeng

United States of America

Thomas Lund

Denmark

Miguel Angel Luque Fernandez

South Africa

Katherine Lust

United States of America

Aleksandra Luszczynska

Poland

May Nawal Lutfiyya

United States of America

Karlen Luthy

United States of America

Eligius Lyamuya

Tanzania

Deborah Lycett

United Kingdom

Courtney Lyles

United States of America

Marita Lynagh

Australia

Cheryl Lynch

United States of America

**AndersMøller**

Sweden

Richard Ma

United Kingdom

Qiaoqin Ma

China

Ruth Mabry

Oman

William MacAllister

United States of America

Georgie MacArthur

United Kingdom

Aristides Machado-Rodrigues

Portugal

Johan Mackenbach

Netherlands

Mhairi Mackenzie

United Kingdom

Mary Mackesy-Amiti

United States of America

Roger Mackett

United Kingdom

Catherine MacPhail

South Africa

Mohsen Maddah

Iran

Hannah Madden

United Kingdom

Purnima Madhivanan

India

Elizabeth Madigan

United States of America

John G. Mæland

Norway

Silje Maeland

Norway

Stefania Maggi

Canada

Dianna Magliano

Australia

Lorenza Magliano

Italy

Pascal Magnussen

Denmark

Michael Johnson Mahande

Tanzania

Pranitha Maharaj

South Africa

Dermot Maher

United Kingdom

Lisa Maher

Australia

Dermot Maher

Uganda

Narayan Maheswary

Bangladesh

Martin Mahoney

United States of America

Xiao-Mei Mai

Norway

Deborah Majerovitz

United States of America

Hoda Makhlouf

Egypt

Tomi Mäkinen

Finland

Robert Ranganai Makombe

Botswana

Michael Makowsky

United States of America

Tayebeh Malek Mohammadi

Iran

Ameer Malik

Pakistan

Vilija Malinauskiene

Lithuania

Davide Malmusi

Spain

Monica Malta

Brazil

Ruslan Malyuta

Switzerland

Simon Henry David Mamuya

Norway

Toshie Manabe

Japan

G. B. John Mancini

Canada

Aliye Mandiracioglu

Turkey

Inacio Mandomando

United States of America

Lindsay Mangham

United Kingdom

Katerina Maniadaki

Grenada

Vijaya Manicavasagar

Australia

Priya Manjoo

Canada

Weerawat Manosuthi

Thailand

Abbas Mansour

Iraq

Ann Manzardo

United States of America

Gian Mauro Manzoni

Italy

Limin Mao

Australia

Myfanwy Maple

Australia

Fiorella Marcellini

Italy

Rick Marchand

Canada

Francois Marclay

Switzerland

Alvin Mares

United States of America

Rodrigo Mariño

Australia

Marc Marí-Dell’Olmo

Spain

Christine Markham

United States of America

Kyriakos Markides

United States of America

Linda Marks

United Kingdom

Elisa Marques

Portugal

Pedro Marques-Vidal

Switzerland

Manuela Marron

Germany

Elliot Marseille

United States of America

Neil Martin

United States of America

Lynne Martin

Australia

Carolyn Martin

United States of America

Miguel Martinez-Gonzalez

Spain

Jose M Martínez-Sánchez

Spain

Vicente Martinez-Vizcaino

Spain

Alexandra Martiniuk

Australia

Regina Martins

Brazil

Florian Marx

Germany

Giovanna Masala

Italy

Irene Misuka Masanja

Tanzania

Bob Mash

South Africa

Saidur Mashreky

Bangladesh

Peninah Masibo

Kenya

Amanda J Mason-Jones

United Kingdom

Mohd Masood

Malaysia

Peter Massey

Australia

Elizabeth Masterson

United States of America

Melanie Matheson

Australia

Flora Matheson

Canada

Catherine Mathews

South Africa

Susana Matias

United States of America

Stefano Mattioli

Italy

Soeren Mattke

United States of America

Christine Mattson

United States of America

Pallab Maulik

India

Webster Mavhu

Zimbabwe

Lindsay Mayberry

United States of America

Elizabeth Rose Mayeda

United States of America

Miguel Angel Mayer

Spain

Mary Mayige

United Kingdom

Olivia Maynard

United Kingdom

Jacek Mazurek

United States of America

Danielle Mazza

Australia

Chidozie Mbada

Nigeria

Blessing Mberu

Kenya

Sheena Mc Hugh

Ireland

Finlay McAlister

Canada

Vivian Mcalister

Canada

Gordon McBean

Canada

Deborah McCahon

United Kingdom

Michelle McCarron

Canada

Mark Mccarthy

United Kingdom

Kerrigan McCarthy

South Africa

Gerry McCartney

United Kingdom

Emma McCartey

United Kingdom

James McCaw

Australia

Joanna McClintock

New Zealand

Rod McCormick

Canada

Tim McCreanor

New Zealand

Louise McCullough

United States of America

Hilary McDermott

United Kingdom

Julie McDonald

Australia

Ted McDonald

Canada

Elizabeth McDonald

Australia

Sharon McDonnell

United States of America

Willi McFarland

United States of America

Patrick McGowan

Canada

Chris McGowin

United States of America

Sarah McIntyre

Australia

Matthew McKenna

United States of America

Eamonn McKeown

United Kingdom

Lindsay McLaren

Canada

Deirdre McLaughlin

Australia

Robert McLeod

Canada

Ruth McNair

Australia

Anna McNulty

Australia

Louise-Anne McNutt

United States of America

Samara McPhedran

Australia

Iain McPhee

United Kingdom

Bruno Meessen

Belgium

Renuka Mehta

United States of America

Jessica Meiklejohn

New Zealand

Wubegzier Mekonnen

Ethiopia

Alemayehu Mekonnen Lemma

Ethiopia

Eivind Meland

Norway

Alfonso Mele

Italy

Alessia Melegaro

Italy

Mengiste Mesfin Melese

Ethiopia

Jaymie Meliker

United States of America

Fiona Mensah

United Kingdom

Gwenn Menvielle

France

Stewart Mercer

United Kingdom

Anwar Merchant

United States of America

Stefano Merler

Italy

Philip Merriam

United States of America

Ray Merrill

United States of America

Arthur Mesas

Spain

Fabio Mesquita

Philippines

Jane Messina

United States of America

Hirohito Metoki

Japan

Christian Meyer

Germany

Philippe Meyer

Switzerland

John D Meyer

United States of America

Elisabeth Meyer

Germany

Charles Mgone

Netherlands

Paola Michelozzi

Italy

Nathalie Michels

Belgium

Nelda Mier

United States of America

Seema Mihrshahi

Australia

Jochen Mikolajczak

Netherlands

Haralampos Milionis

Greece

Jose Mill

Brazil

Arlene Miller

United States of America

Michael Miller

United States of America

Laurence Millon

France

Brennen Mills

Australia

Samuel Mills

United States of America

Julie Milstien

France

Adrian Mindel

Australia

Jennifer Mindell

United Kingdom

J. Jaime Miranda

Peru

Anoop Misra

India

Ritesh Mistry

United States of America

Ellen M.H. Mitchell

Netherlands

Pamela Mitchell

United States of America

Jan Mitosinka

Slovakia

Sophie Mitra

United States of America

Kyoko Miura

Australia

Elia John Mmbaga

Tanzania

Tomas Moberg

Sweden

Chawangwa Modongo

Botswana

Sepideh Modrek

United States of America

Bente Moen

Norway

Vittal Mogasale

Korea South

Manju Mohan

India

Yashar Moharamzad

Iran

Sigrid M. Mohnen

Netherlands

Anu Molarius

Sweden

Michal Molcho

Ireland

NoelleAngelique Molinari

United States of America

Nicolas Molinari

France

Diego Moliner-Urdiales

Spain

Yordanos Belayneh Molla

Ethiopia

Liesbeth Mollema

Netherlands

Willie Mollentze

South Africa

Ute Mons

Germany

Julio Montaner

Canada

Ali Montazeri

Iran

Douglas Monteiro

Brazil

Catherine Montgomery

United Kingdom

Kotsedi Daniel Monyeki

South Africa

Graham Moon

United Kingdom

Mauro Batista Morais

Brazil

Melinda Moree

United States of America

Marina Moreira

Brazil

Pedro Moreira

Portugal

Carla Moreira

Portugal

Meade Morgan

United States of America

Philip Morgan

Australia

Shingo Moriya

Japan

Bjørn Mørkedal

Norway

Olawale Moronkola

Nigeria

Meghan Morris

United States of America

Barbara Morrongiello

Canada

Ole Hartvig Mortensen

Denmark

Hanns Moshammer

Austria

Howard Moss

United States of America

Golam Mostofa

Bangladesh

Yutaka Motohashi

Japan

Jin Mou

Canada

Anne Moyer

United States of America

Philippe Msellati

Burkina Faso

Kelias Msyamboza

Malawi

Alison Mudge

Australia

Joan Muela Ribera

Spain

Edgar Mueller

Germany

Peter Muennig

United States of America

Kathryn Muessig

United States of America

Anthony Mugisha

Uganda

Dieudonne Muhoza

Rwanda

Matt Muijen

Denmark

Elizabeta Mukaetova-Ladinska

United Kingdom

Cornelis Mulder

Netherlands

Katherine Muldoon

Canada

Luise Müller

Denmark

Dirk Müller

Germany

Sarah Munce

Canada

Fehmidah Munir

United Kingdom

Carles Muntaner

Canada

Yoshitaka Murakami

Japan

Shigeyuki Muraki

Japan

Hiroshi Murayama

United States of America

Joan Murphy

United Kingdom

Dean Murphy

Australia

Debra Murphy

United States of America

Padmini Murthy

United States of America

David Muscatello

Australia

Nyokabi Musila

Kenya

Adamson Sinjani Muula

Malawi

Adamson Muula

Malawi

Mark Myatt

United Kingdom

Arnstein Mykletun

Norway

Huseyin Naci

United Kingdom

Toru Nagao

Japan

Gabriele Nagel

Germany

Mohsen Naghavi

United States of America

Devaki Nair

United Kingdom

Tomoko Nakagami

Japan

Hiroyuki Nakamura

Japan

Yasuhide Nakamura

Japan

Yuko Nakao

Japan

Akinori Nakata

United States of America

Jolly Nankunda

Uganda

Punithavathi Narayanan

Malaysia

Masa Narita

United States of America

Kulaya Narksawat

Thailand

Denis Nash

United States of America

Aashir Nasim

United States of America

George Nassis

Greece

Javaid Nauman

Norway

Kumari Navaratne

Sri Lanka

Ana Navas-Acien

United States of America

Patti-Jean Naylor

Canada

David Ndetei

Kenya

Mzikazi Nduna

South Africa

Saharnaz Nedjat

Iran

Lee Nedkoff

Australia

Johanna Helena Nel

South Africa

Toben Nelson

United States of America

Paul Nelson

Australia

Julianna Nemeth

United States of America

Tooru Nemoto

United States of America

Kara Nerenberg

Canada

Andrea Nesbitt

Canada

Daniel Nettle

United Kingdom

Christopher Nettles

United States of America

Cory Neudorf

Canada

Lynnette Neufeld

Canada

Dianne Neumark-Sztainer

United States of America

Heike Neumeister-Kemp

Australia

Dorothy Newbury-Birch

United Kingdom

Lori Newman

Switzerland

Joshua Daniel Newton

Australia

Sam Newton

Ghana

Edward Ng

Canada

Shu Wen Ng

United States of America

Nawi Ng

Sweden

Shirley P.C. Ngai

Hong Kong

Ha Nguyen

United States of America

Gordon Nichols

United Kingdom

Melanie Nichols

Australia

Theresa Nicklas

United States of America

Sophie Nicklaus

France

Nicola Nicolotti

Italy

Jesper Bo Nielsen

Denmark

Anne Nielsen

Denmark

Rune Nielsen

Norway

Morten Birkeland Nielsen

Norway

Karina Nielsen

Denmark

Steffen Niemann

Switzerland

Pentti Nieminen

Finland

Tilahun Nigatu

Australia

Béatrice Nikiema

Canada

Louise Nilunger Mannheimer

Sweden

Feng Ning

Finland

Kaijun Niu

Japan

Bernard Njau

Tanzania

Douglas Noble

United Kingdom

Iskra Alexandra Nola

Croatia

Abdisalan M Noor

Kenya

Mikael Nordenmark

Sweden

Paul Norman

United Kingdom

Gregory Norman

United States of America

Marie Norredam

Denmark

Carol North

United States of America

John Noti

United States of America

Scott P. Novak

United States of America

Mary Patricia Nowalk

United States of America

Zoann Nugent

Canada

Rachel Nugent

United States of America

Baltazar Nunes

Portugal

Fred Nuwaha

Uganda

Obioma Nwaorgu

Nigeria

Myo Nyein Aung

Thailand

Karin Nygard

Norway

Maria Nyholm

Sweden

Carol Oatis

United States of America

Isidore Obot

Nigeria

Oonagh O’Brien

United Kingdom

Brigit Obrist

Switzerland

Sean O’Cathail

United Kingdom

Matjaz Ocepek

Slovenia

Sophie Ochola

Kenya

Dianne O’Connell

Australia

Elaine O’Connell

United Kingdom

Wendy Oddy

Australia

Andrew Odegaard

United States of America

Melissa O’Donnell

Australia

Olumuyiwa Odusanya

Nigeria

Olumuyiwa Odusanya

Nigeria

John Oeltmann

United States of America

Eyitope Ogunbodede

Nigeria

Tinuade Ogunlesi

Nigeria

Ross O’Hara

United States of America

Henrik Ohlsson

Sweden

Heather Ohly

United Kingdom

Kristiina Ojala

Finland

Kazue Okamoto-Mizuno

Japan

James Okello

Uganda

Amolo Okero

Switzerland

Tuula Oksanen

United States of America

Tuula Oksanen

Finland

Bolanle Ola

Nigeria

Amos Olagunju

United States of America

Ellinor Olander

United Kingdom

Sean O’Leary

United States of America

Caroline O’Leary

United Kingdom

Manuel Olivares

Chile

Jake Olivier

Australia

Lemessa Oljira

Ethiopia

Lénaïck Ollivier

France

Niclas Olofsson

Sweden

Jennifer O’Loughlin

Canada

Ashley Olson

United Kingdom

Dana Lee Olstad

Canada

Sepideh Omidvari

Iran

Altan Onat

Turkey

David Ong

Netherlands

Hans Onya

South Africa

Eline Op de Coul

Netherlands

Don Operario

United States of America

Carlos Orces

United States of America

Nicoletta Orchi

Italy

Kevin O’reilly

Switzerland

Heather Orom

United States of America

Peter O'Rourke

Australia

Giovanni Battista Orsi

Italy

Richard Osborne

Australia

Frank Osei

Ghana

Yewande Oshodi

Nigeria

Joanne O’Toole

Australia

Emilio Ovuga

Uganda

Lesley Owen

United Kingdom

Eme Owoaje

Nigeria

Ellis Owusu-Dabo

United Kingdom

Andy Oxman

Norway

Kola Oyediran

Nigeria

Adewale Oyeyemi

Nigeria

Levent Özdemir

Turkey

Sukru Ozen

Turkey

Marja Pöllänen

Finland

Laura Paalanen

Finland

Gisel Padula

Argentina

Fabrice Paganin

France

Andrew Page

Australia

Riitta Pahkala

Finland

Soon-Young Paik

Korea South

Pascal Paillé

Canada

Vera Paiva

Brazil

Gianni Pala

Italy

Krishna Mohan Palipudi

United States of America

Ståle Pallesen

Norway

Antonio Palmeira

Portugal

Jia-Yan Pan

Hong Kong

Chien-Yu Pan

Taiwan

Kaushik Pandit

India

Massimiliano Panella

Italy

Pinaki Panigrahi

United States of America

Jenna Panter

United Kingdom

Angeliki Papadaki

Greece

Nikolaos Papanas

Greece

George Papathanasiou

Greece

Constantina Papoutsakis

Greece

Evelina Pappa

Greece

Tiago Paredes

Portugal

Sailajanandan Parida

India

Manmohan Parida

India

Subhash Parija

India

Mi Jung Park

Korea South

Lynne Parkinson

Australia

Diana Parra

United States of America

Anne-Maree Parrish

Australia

Keryn Pasch

United States of America

Paige Passano

United States of America

Jonathon Passmore

Viet Nam

Valeria Passos

Brazil

Bev Paterson

Australia

Thomas L Patterson

United States of America

Abel Pavia

Mexico

Ana Luiza Braz Pavão

Brazil

John Peabody

United States of America

Anna Pearce

United Kingdom

Natalie Pearson

United Kingdom

Jennifer Pearson

United States of America

Jane Pearson

United States of America

Linda Pederson

United States of America

Linda Pederson

Canada

Mangesh Pednekar

India

Eric Walter Pefura Yone

Cameroon

Camille Pelat

France

Christopher Pell

Netherlands

Markku Peltonen

Finland

Karl Peltzer

South Africa

Andrea Barnabas Pembe

Tanzania

Claire Penn

South Africa

Linda Penn

United Kingdom

Julian Perelman

Portugal

Rom Perenboom

Netherlands

Marco Peres

Brazil

Armando Perez-Cueto

Denmark

Rafael Perez-Escamilla

United States of America

Ana B Pérez-Lizaur

Mexico

Gastón Perman

Argentina

Laure Perrier

Canada

Richard Peter

Germany

Janina Petkeviciene

Lithuania

Paul Petraro

United States of America

Tom Pettersson

Finland

Stefano Petti

Italy

Audrey Pettifor

United States of America

Simone Pettigrew

Australia

Lorenzo Pezzoli

Switzerland

Patrizio Pezzotti

Italy

Davies Mubika Pfukenyi

Zimbabwe

Carl Phillips

Canada

Kristina Phillips

United States of America

Hwee Pin Phua

Singapore

Mercedes Pico

Argentina

Eduardo David Piemonte

Argentina

Zuzanna Pieniak

Belgium

Kirsi Pietiläinen

Finland

Mark Pietroni

United Kingdom

Michael Pignone

United States of America

Ian Pike

Canada

Hynek Pikhart

United Kingdom

Terri Pikora

Australia

Harold Pincus

United States of America

Ilana Pinsky

Brazil

Roberta Pirastu

Italy

Jane Pirkis

Australia

Catherine Pirkle

Canada

Paola Pisani

Italy

Christophe Pison

France

Christos Pitsavos

Guam

Andreia Nogueira Pizarro

Portugal

Sandra Plachta-Danielzik

Germany

Inger Plaisier

Netherlands

Rebeca Plank

United States of America

Megan Pledger

New Zealand

Ozren Polasek

Croatia

Tamara Poljicanin

Croatia

Keshia Pollack

United States of America

Sue Pollock

Canada

Kevin Pollock

United Kingdom

Laura Pomeroy

United States of America

Maurizio Pompili

Italy

Sujata Ponappa

United States of America

Constance Dimity Pond

Australia

Audrey Poon

Canada

Barry Popkin

United States of America

Svetlana Popova

Canada

Kholoud Porter

United Kingdom

Judy Porter

United States of America

Ann Post

Sweden

Maarten Postma

Netherlands

Julia Potter

United Kingdom

Michelle Poulin

United States of America

Janet Powell

United Kingdom

Thomas Power

United States of America

Elaine Power

Canada

Krishnananda Prabhu

India

Jetsumon Prachumsri

Thailand

Rosa Prato

Italy

José Precioso

Portugal

Michèle Preyde

Canada

Matt Price

United States of America

Anna Price

Australia

Charlotte Price

United Kingdom

Anna-Eva Prick

Netherlands

Naomi Priest

Australia

Mary Pritchard

United States of America

Susan Proctor

United States of America

Limthong Promdee

Thailand

Paul Pronyk

United States of America

Paul Pronyk

United States of America

Joanne Protheroe

United Kingdom

Christof Prugger

France

Gabrielle Pucci

Brazil

Giuseppe Pugliese

Italy

Joan Puig-Barberà

Spain

Eleanor Pullenayegum

Canada

Daryl Pullman

Canada

Thomas Pullum

United States of America

Vincenzo Puro

Italy

Edward Purssell

United Kingdom

Pekka Puska

Finland

Elisa Puzzolo

United Kingdom

Shazia Qasim

Malaysia

Harith Qazaz

Iraq

liqian Qiu

China

Jon Quach

Australia

Xavier Quantin

France

John Quillin

United States of America

Micheal Quinlan

United Kingdom

Micheal Quinlan

Australia

Rocio Quinonez

United States of America

Penelope J Quintana

United States of America

Frances Quirk

Australia

Pham Quynh

Australia

Susan Racette

United States of America

Carmen Radecki Breitkopf

United States of America

Shafquat Rafiq

United Kingdom

Alberto Raggi

Italy

Saifur Rahman

Australia

Pavani Ram

United States of America

Ambady Ramachandran

India

Rohit Ramaswamy

United States of America

Rajeev Ramchand

United States of America

A Susana Ramirez

United States of America

Lynn Ramirez

United States of America

Danielle Ramo

United States of America

Jose Manuel Ramos

Spain

Sarah Randolph

United Kingdom

Merja Rantakokko

Finland

Dennis Raphael

Canada

Mitra Rashidian

Australia

Daniel Rasic

Canada

Eva-Britta Råssjö

Sweden

Nilantha Ratnayake

Sri Lanka

Elena Ratschen

United Kingdom

Tobias Raupach

Germany

Ulrike Ravens-Sieberer

Germany

Rebecca Ray

United States of America

Deborah Lynn Reas

Norway

Matejka Rebolj

Denmark

Gabriel Reboux

France

Cassiano Ricardo Rech

Brazil

Anthony Redmond

United Kingdom

Josep Redon

Spain

Erin Rees Clayton

United States of America

Katherine Reeves

United States of America

Enrique Regidor

Spain

Michael Regier

United States of America

Eva Rehfuess

Germany

David Rehkopf

United States of America

Nicholas Reich

United States of America

Michael Reichenheim

Brazil

Felipe Reichert

Brazil

Jan Reinhardt

Switzerland

Belinda Reininger

United States of America

Harald Reiso

Norway

Silje Endresen Reme

United States of America

Lillian Remer

United States of America

Aiguo Ren

China

Tamar Renaud

United States of America

Andrew Renehan

United Kingdom

Mary Renfrew

United Kingdom

Elisha Renne

United States of America

Andre Renzaho

Australia

Stella Resko

United States of America

David Resnik

United States of America

Grégoire Rey

France

Hugh Reyburn

United Kingdom

Humberto Reynoso

United States of America

Fernando Ribeiro

Portugal

Dominique Ricard

Laos

Brian Rice

United Kingdom

Brian Rice

South Africa

Ira Richards

United States of America

Justin Richards

United Kingdom

Amanda Richardson

United States of America

Nicola Diane Ridgers

Australia

Ken Rigby

Australia

Matthew Rimmer

Australia

Lionel Riou França

France

Heleen Riper

Netherlands

Josien Riphagen-Dalhuisen

Netherlands

Laetitia Rispel

South Africa

Chris Rissel

Australia

Matti Ristola

Finland

Catherine Ritter

Switzerland

Raphael Ritti-Dias

Brazil

Jose Rizzo

Brazil

Nico Rizzo

United States of America

Assegid Roba

Mozambique

Gail Robinson

Canada

Chris Robinson

Canada

Suzan Robroek

Netherlands

Evangelista Rocha

Portugal

Donna Roche

Canada

Peter Rockers

United States of America

Ian Rockett

United States of America

Sarah Rodgers

United Kingdom

Luis Rodrigues

Portugal

Fernando Rodríguez-Artalejo

Spain

Keetie Roelen

United Kingdom

Corne Roelen

Netherlands

Karolin Roeser

Germany

Ann Roex

Belgium

Elizabeth Rogawski

United States of America

Anne Elizabeth Rogers

United Kingdom

Christine Rogers

United States of America

Nigel Rollins

South Africa

Dan Romer

United States of America

Andrea Romero

United States of America

Roberto Romi

Italy

Thomas Romig

Germany

shuang Rong

China

Telmo Ronzani

Brazil

Eira Roos

Finland

Eva Roos

Finland

Annina Ropponen

Finland

Nelson Rosario

Brazil

Vanessa Rose

Australia

Michael Rosenberg

Australia

Philip Rosoff

United States of America

Robert Ross

Canada

Hana Ross

United States of America

Annalisa Rosso

Italy

Gergely Röst

Hungary

Matteo Rota

Italy

Ulrike Rothe

Germany

Emily Rothman

United States of America

Mark Rothstein

United States of America

John Arne Rottingen

Norway

Matthew Rousu

United States of America

Richard Rowe

United Kingdom

Vsevolod Rozanov

Ukraine

R Gary Rozier

United States of America

Yuhua Ruan

China

Anna Rubinsky

United States of America

Nancy Rudner

United States of America

Montserrat Rue

Spain

Vincent Rue

United States of America

Reiner Rugulies

Denmark

Krassi Rumchev

Australia

Christina Rundi

Malaysia

Mark Russi

United States of America

Luis Rustveld

United States of America

Lainie Rutkow

United States of America

Tom Rutledge

United States of America

Carolyn Rutter

United States of America

Femke Rutters

United Kingdom

Carrie Ruxton

United Kingdom

Soheil Saadat

Iran

Osmo Saarelma

Finland

Charumathi Sabanayagam

Singapore

Heba Sabbagh

Senegal

Keith Sabin

Viet Nam

Harshpal Sachdev

India

Ulrich Sack

Germany

David Sack

United States of America

Gary Sacks

Australia

Rachel Sacks-Davis

Australia

Klas-Goran Sahlen

Sweden

Rihlat Said-Mohamed

South Africa

Pedro Saint-Maurice

United States of America

Isao Saito

Japan

Kabiru Salami

Nigeria

Mariano Salazar

Sweden

Sarah Saleem

Pakistan

Amin Salehi Abargouei

Iran

Mo Salman

United States of America

Simo Salminen

Finland

Ferdinand Salonna

Czech Republic

Kjell Salvesen

Norway

Francisco Mauro Salzano

Brazil

Sharon Samet

United States of America

Florence Samkange-Zeeb

Germany

Julia Samuelson

Switzerland

Miguel San Sebastian

Sweden

Zila Sanchez

Brazil

Travis Sanchez

United States of America

Sergio Sanchez-Garcia

Colombia

Mairena Sanchez-Lopez

Spain

Antoinette Sander

United States of America

Sara Sanders

United States of America

Helene Sandmark

Sweden

Rassamee Sangthong

Thailand

Dia Sanou

Canada

Augusto Hasiak Santo

Brazil

Rute Santos

Portugal

Itamar Santos

Brazil

Diana Santos

Portugal

Simone Santos

Brazil

Maria Paula Santos

Portugal

Ana Santos

Portugal

Valeria Saraceni

Brazil

Sarita Sardana

India

JItender Sareen

Canada

Ginny Sargent

Australia

Mary Saridi

Greece

Olga Sarmiento

Colombia

Rosella Saulle

Italy

Judith Savageau

United States of America

Sharon Saydah

United States of America

Carmen Sayon-Orea

Spain

Luciana Scalone

Italy

Justin Scanlan

Australia

Alberto Scarselli

Italy

Marcia Scazufca

Brazil

Julius Schachter

United States of America

Christian Schaetti

Switzerland

Dena Schanzer

Canada

Ted Scharf

United States of America

Jason Schaub

United States of America

Frieder Schaumburg

Germany

Edward Schelegle

United States of America

Niels Schenk

Netherlands

Jean Schensul

United States of America

W. William Schluter

Nepal

Axel J Schmidt

Germany

Wolf-Peter Schmidt

United Kingdom

Reinhard Schnettler

Germany

Ulrich Schnyder

Switzerland

Monica Schoch-Spana

United States of America

Shaun Scholes

United Kingdom

Ronaldo Scholte

Brazil

Bert Schreuder

Netherlands

Ingrid Schubert

Germany

Kathrin Schuck

Netherlands

Dena Schulman-Green

United States of America

Andreas Schulte

Germany

Jörg Schüpbach

Switzerland

Merel Schuring

Netherlands

Marlene Schwartz

United States of America

Peter Schwarz

Germany

Eli Schwarz

United States of America

Florian J Schweigert

Germany

Kent Schwirian

United States of America

Anthony Scialli

United States of America

John Scott

Australia

Lori Scott-Sheldon

United States of America

Jamie Seabrook

Canada

Holly Seale

Australia

Andrew Searles

Australia

Rene Sebena

Slovakia

Sanaz Sedaghat

Netherlands

Laurence Seematter

Switzerland

Jeremy Segrott

United Kingdom

Jorma Seitsamo

Finland

Randi Selmer

Norway

Dyanne Semogas

Canada

Thomas Semrad

United States of America

Bisakha Sen

United States of America

Ibrahim Sendagire

Uganda

Ian Seppelt

Australia

Francesco Sera

Italy

Berrin Serdar

United States of America

Ajay Sethi

United States of America

Maninder Singh Setia

India

Gianluca Severi

Australia

Vikash Sewram

South Africa

Muhammad Aslam Shad

Pakistan

Omar Shafey

United Arab Emirates

Stephen Shafran

Canada

Baiju Shah

Canada

Syed Ghulam Sarwar Shah

United Kingdom

Akhawri Shankar

India

Steven Shapiro

United States of America

Robert Shapiro

United States of America

Estifanos Biru Shargie

Switzerland

Joseph Sharkey

United States of America

Sangita Sharma

Canada

Mukta Sharma

Thailand

William Shaw

United States of America

Keiba Shaw

United States of America

Janet Shaw

United States of America

Brenna Shearer

Canada

Aubrey Sheiham

United Kingdom

Kristen Shellenberg

United States of America

Xiao-Yang Sheng

China

Jessica Sheringham

United Kingdom

Zumin Shi

Australia

Yipu Shi

Canada

Ju-Fang Shi

Australia

Ling Shi

United States of America

Zumin Shi

Australia

Meredith Shiels

United States of America

Frances Shiely

Ireland

Ruth Shim

United States of America

Aesun Shin

Korea South

Tomohiro Shinozaki

Japan

Rosalyn Shute

Australia

Arjumand Siddiqi

Canada

Nazish Siddiqi

United States of America

Johannes Siegrist

Germany

Dagmar Sigmundová

Czech Republic

Tadesse Siisay

Ethiopia

Bernard Silke

Ireland

Paula Silva

Portugal

Gustavo Silva

Portugal

Diego Silva

Brazil

Marc Silverstein

United States of America

Laura Sima

United States of America

Padam Simkhada

United Kingdom

Cory Simon

Canada

Vanessa Simonds

United States of America

Jaromír Simonek

Slovakia

Leonardo Simonella

Singapore

Kavita Singh

United States of America

Bhanu Sinha

Netherlands

John Sirard

United States of America

Susan Sisson

United States of America

Sheila Skeaff

New Zealand

Steven Skov

Australia

Patty Skuster

United States of America

Linda Slack-Smith

Australia

Gary Slade

Australia

Martin Slade

United States of America

Adrian Sleigh

Australia

Diewertje Sluik

Germany

Rhonda Small

Australia

Neil Smart

Australia

Beitske Smink

Netherlands

Colette Smit

Netherlands

Elaine Smith

United States of America

Albert Smith

United States of America

Kimberley Smith

Canada

Debbie Smith

United Kingdom

Ben J Smith

Australia

Helen J Smith

United Kingdom

Lisa Smith

United States of America

Neil Smith

United Kingdom

Katherine Smith

United States of America

Ruth Smith

United Kingdom

Megan Smith

Australia

Neale Smith

Canada

Lucy Smith Paintain

United Kingdom

Kate Smolina

United Kingdom

Bobby Smyth

Ireland

John Snelgrove

Canada

Karla Soares-Weiser

United Kingdom

Stéphane Sobczak

United States of America

Maja Socan

Slovenia

Marianna Sockrider

United States of America

Bjorn Soderfeldt

Sweden

Remko Soer

Netherlands

Maria Giuliana Solinas

Italy

Hans Magnus Solli

Norway

Tewarit Somkotra

Thailand

Egbert Sondorp

United Kingdom

Anna Song

United States of America

Yun-Mi Song

Korea South

John Song

United States of America

Elijah Songok

Kenya

Susan Sonne

United States of America

Kusol Soonthorndhada

Thailand

Hamid Soori

Iran

Francesco Sopracordevole

Italy

Kristine Sorensen

Netherlands

Corinna Sorenson

United Kingdom

Enrique R. Soriano

Argentina

Alberto Soriano-Maldonado

Spain

Bundit Sornpaisarn

Canada

Zvonko Sosic

Croatia

Elpidoforos Soteriades

Cyprus

Emily Sousa

United States of America

Maria Alice Souza de Oliveira Dode

Brazil

Dionysios Spanopoulos

United Kingdom

Natalie Spearing

Australia

John Spence

Canada

Brenda Spencer

Switzerland

Matthew Sperrin

United Kingdom

Joanne Spetz

United States of America

Jeffery Spickett

Australia

Anneliese Spinks

Australia

Frank Spradley

United States of America

Andrew Springer

United States of America

Stephen Bertel Squire

United Kingdom

Chandrashekhar Sreeramareddy

Nepal

Penpatra Sripaiboonkij

Thailand

Patcharawan Srisilapanan

Thailand

Freddie Ssengooba

Uganda

Mandy Stahre

United States of America

Amanda Staiano

United States of America

Carin Staland-Nyman

Sweden

Marie Standl

Germany

Debbi Stanistreet

United Kingdom

Rosemary Stanton

Australia

Victoria Staples

United Kingdom

Gregor Starc

Slovenia

Klaus Stark

Germany

Renee Stark

Germany

Afroditi Stathi

United Kingdom

Marc Steben

Canada

Andrew Steer

Australia

Cheryl R Stein

United States of America

Julia Steinberg

United States of America

Laura Steinhardt

United States of America

Amy Steinkellner

United States of America

Peter Steinmann

Switzerland

Aslak Steinsbekk

Norway

Ethelwynn Linda Stellenberg

South Africa

Marlene Stenbacka

Sweden

Stacie Stender

South Africa

Benedicte Stengel

France

Mark Stenger

United States of America

Niamh Stephenson

Australia

Alexandra Stewart

United States of America

Mary Stewart

Australia

Kelton Stewart

United States of America

Jennifer Stewart Williams

Australia

Melissa Stigler

United States of America

Marija Stipic

Croatia

Christiane Stock

Denmark

Emily Stockings

Australia

Wolfgang Stöhr

United Kingdom

Marijn Stok

Netherlands

Edita Stokic

Serbia

Manfred Stommel

United States of America

Ingunn Størksen

Norway

Mark Storrs

Australia

Kristi Storti

United States of America

Michael Stoto

United States of America

Mattias Strandh

Sweden

Saverio Stranges

United Kingdom

Ruth Striegel-Moore

United States of America

Jorien Emelien Strijk

Netherlands

Silvia Stringhini

Switzerland

Ralf Strobl

Germany

Patricia Struthers

South Africa

Heather Stuart

Canada

Andreas Stuck

Switzerland

David Stuckler

United Kingdom

Cynthia Stuhlmiller

Australia

Jackie Sturt

United Kingdom

Linyan Su

China

Parminder Suchdev

United States of America

Haruhiko Sugimura

Japan

Takemi Sugiyama

Australia

Nabil Sulaiman

United Arab Emirates

Markku Pertti Tapio Sumanen

Finland

Athula Sumathipala

United Kingdom

Walton Sumner

United States of America

Wei Sun

China

Lei Sun

China

Anne Mari Sund

Norway

Gerhard Sundborn

New Zealand

Johanne Sundby

Norway

Josefin Sundin

United Kingdom

Hai-Yen Sung

United States of America

Tarja Suominen

Finland

Sakari Bertel Alfred Suominen

Finland

Rajah Supramaniam

Australia

Xisca Sureda Llull

Spain

Chalida Svastisalee

Denmark

Ann Swartz

United States of America

Dallas Swendeman

United States of America

Boyd Swinburn

New Zealand

Mieczyslaw Szyszkowicz

Canada

Yehualashet Tadesse

Ethiopia

Miriam Taegtmeyer

United Kingdom

Tiffany Taft

United States of America

Harry Tagbor

Ghana

Nina Tahhan

Australia

Tim K Takaro

Canada

Eiji Takeda

Japan

Misa Takegami

Japan

Mohammad Talaei

Iran

Sameera Talegawkar

United States of America

Haiping Tan

Australia

Deliang Tang

United States of America

Wenjie Tang

China

Viroj Tangcharoensathien

Thailand

Arunrat Tangmunkongvorakul

Thailand

Cetin Tanrikulu

Turkey

Pierre Tattevin

France

Hermano Tavares

Brazil

Anne Taylor

Australia

Douglas Taylor

United States of America

C. Barr Taylor

United States of America

Lee Taylor

Australia

Mark Taylor

United Kingdom

David Taylor-Robinson

United Kingdom

Anastase Tchicaya

Luxembourg

Fasil Tekola

Ethiopia

Richard Telford

Australia

Shirley Telles

India

Elizabeth Temkin

Israel

Iva Tendais

Portugal

Cheong Lieng Teng

Malaysia

Edmond Teng

United States of America

Eric Tenkorang

Canada

Marc Tennant

Australia

Ruth Tennant

United Kingdom

Claudia Terschüren

Germany

Fessahaye Tesfamichael

Ethiopia

Fikru Tesfaye

Ethiopia

Beatriz H Tess

Brazil

Megan Teychenne

Australia

Sarah Thackway

Australia

Thaksaphon Thamarangsi

Thailand

Prashanth Thankachan

India

Narbada Thapa

Nepal

Kevin Theunissen

Netherlands

Hélène Thibault

France

Hanne Thiede

United Kingdom

Matthew Thiese

United States of America

Betsy Thom

United Kingdom

Heather Clarke Thomas

Canada

Beena Thomas

India

Roger Thomas

Canada

David Thomas

Australia

Lisa Thompson

United States of America

William Murray Thomson

New Zealand

Bruce Thorley

Australia

Claire Thorne

United Kingdom

Andrew Thorne-Lyman

United States of America

Simon Thornley

New Zealand

Lukar Thornton

Australia

James Thrasher

United States of America

Miranda Thurston

United Kingdom

Lau Thygesen

Denmark

Hao Tian

United States of America

Martin Tickle

United Kingdom

Tanongson Tienthavorn

Thailand

Tanongson Tienthavorn

Thailand

Ans H Tiessen

Netherlands

Hong Van Tieu

United States of America

Marjaana Tikanmäki

Finland

Benedikt Till

Austria

Therese Tillin

United Kingdom

Aura Timen

Netherlands

Hilary Tindle

United States of America

Sarah Ting

United States of America

Bjorn Tingberg

Sweden

Sylvia Titze

Austria

Guy Tobias

Israel

Martin Tobias

New Zealand

Catherine Todd

United States of America

Susanna Toivanen

Sweden

Estefania Toledo

Spain

Janne Tolstrup

Denmark

Mimmi Tolvanen

Finland

Van Tong

United States of America

Shilu Tong

Australia

Kjell Torén

Sweden

Paul Torgerson

Switzerland

Essie Torres

United States of America

Rosa J Torres

Spain

Sebahat Dilek Torun

Turkey

Igor Toskin

Switzerland

Loraine Townsend

South Africa

Alberto Tozzi

Italy

Thach Tran

Viet Nam

Paul Tranter

Australia

Lyndal Trevena

Australia

Daisson Trevisol

Brazil

Maria Trigoni

Greece

Jenny Trinitapoli

United States of America

Elisa Trucco

United States of America

Jack Tsai

United States of America

Georgios Tsakos

United Kingdom

Apostolos Tsapas

Greece

Aster Tsegaye

Ethiopia

Chunchieh Tseng

Taiwan

Komla Tsey

Australia

Fatima Tsiouris

United States of America

Kenji Tsunoda

Japan

Mary Tuba

Zambia

Reginald Tucker-Seeley

United States of America

Catrine Tudor-Locke

United States of America

Olivia Tulloch

United Kingdom

Alex Tulloch

United Kingdom

Helena Tunstall

United Kingdom

Akif Turna

Turkey

Deborah Turnbull

Australia

Katy Turner

United Kingdom

Gavin Turrell

Australia

Lisa Tussing-Humphreys

United States of America

Aage Tverdal

Norway

Jos Twisk

Netherlands

Wu Ty

China

Flora Tzelepis

Australia

Dong-Sheng Tzeng

Taiwan

Konstantinos Tziomalos

Greece

Maduka Ughasoro

Nigeria

Donatella Ugolini

Italy

Kingsley Nnanna Ukwaja

Nigeria

Jennifer Unger

United States of America

Brigid Unim

Italy

Krishna Upadhya

United States of America

Lianne Urada

United States of America

Karen Urbanoski

Canada

Olalekan Uthman

Niger

William Vásquez

United States of America

Jose Lorenzo Valencia-Martin

Spain

Marta Valenciano

Spain

Andrew Vallely

Australia

Donna Vallone

United States of America

Pieter HM van Baal

Netherlands

Ed van Beeck

Netherlands

Ellen Van de Poel

Netherlands

Ingrid Viola Francine van den Broek

Netherlands

Ingrid van der Mei

Australia

Monique van der Veen

Netherlands

Philip Van der Wees

Netherlands

Marieke J. van der Werf

Sweden

Tjip van der Werf

Netherlands

Marieke J. van der Werf

Netherlands

Jouke van der Zee

Netherlands

Anna Maria van Eijk

Ethiopia

Jean-Pierre Van geertruyden

Belgium

Marleen van Gelder

Netherlands

Ann Van Hecke

Belgium

Geneviève van Liere

Netherlands

Esther Van Lieshout

Netherlands

Francois van Loggerenberg

United Kingdom

Harm van Marwijk

Netherlands

Erik Van Miert

Belgium

Rudolph Leon van Niekerk

South Africa

Hein van Onzenoort

Netherlands

Herman Van Oyen

Belgium

Eric van Rongen

Netherlands

Heidi van Rooyen

South Africa

Joost van Rosmalen

Netherlands

Ronan Van Rossem

Belgium

Esther van Sluijs

United Kingdom

Liza van Steenbergen

Netherlands

Irene van Valkengoed

Netherlands

Mariska van Vliet

Netherlands

Nicholas Van Wagoner

United States of America

Elizabeth VanDenKerkhof

Canada

Robert Vander Stichele

Belgium

Jacques Vanobbergen

Belgium

Apostolos Vantarakis

Greece

Jeffrey VanWormer

United States of America

Maria Inês Varela-Silva

United Kingdom

Ebrahim Variava

South Africa

Juha Varrela

Finland

Juan Vasconez

Ecuador

Amber Vaughn

United States of America

Dorothy Vawter

United States of America

Tord Finne Vedoy

Norway

Sargoor Veena

India

Ruut Veenhoven

Netherlands

Lennert Veerman

Australia

Paula Veiga

Portugal

Jenny Veitch

Australia

Inés Velasco

Spain

Gustavo Velásquez-Melendez

Brazil

Danae Venieri

Greece

Maria Amelia Veras

Brazil

Marie-Noël Vercambre

France

Petra Verdonk

Netherlands

Nick Verhaeghe

Belgium

Eduard Verhagen

Netherlands

Arpana Verma

United Kingdom

Angelique Vermeiren

Netherlands

Ad Vermulst

Netherlands

Sten Vermund

United States of America

Michel Vernay

France

Annette Verster

Italy

Suzanne Verver

Netherlands

Nicolas Veziris

France

Benjamin Vicente

Chile

German Vicente-Rodriguez

Spain

Josep Vidal

Spain

Alejandro Videla

Argentina

Tapio Videman

Canada

Damon Vidrine

United States of America

Rachel Vieux

France

Jana Vignerova

Czech Republic

Maria Pia Villa

Italy

Salvador Villalpando

Mexico

Andrea Villanti

United States of America

Karen Villanueva

Australia

Deborah Vincent

United States of America

Eva Vingård

Sweden

Annika Viniol

Germany

Concepcion Violan-Fors

Spain

Jorma Virtanen

Finland

Pekka Virtanen

Finland

Marianna Virtanen

Finland

Javier Virués-Ortega

Canada

Leela Visaria

India

Charlotte Vissenberg

Netherlands

Maretha Visser

South Africa

Mohan Viswanathan

India

Gopalan Viswanathan

India

Francesco Vitale

Italy

Alejandra Vives

Chile

Charalambos Vlachopoulos

Greece

Line Vold

Norway

Albert Vollaard

Netherlands

Thomas von Lengerke

Germany

Tido von Schoen-Angerer

Switzerland

Rimke Vos

Netherlands

Panos Vostanis

United Kingdom

Anne Vuillemin

France

Eugene Waclawski

Canada

Tracey Wade

Australia

Yasir Waheed

Pakistan

Mark Wahlqvist

Taiwan

Gabriel Waisman

Argentina

Peter Waiswa

Uganda

Melanie Wakefield

Australia

David Walker

United Kingdom

Jennifer Walker

Australia

Karen Walker

Australia

Martin Wall

New Zealand

Lorraine Wallace

United States of America

Susan Wallace

United Kingdom

Jo Waller

United Kingdom

Thorne Wallman

Sweden

Helen Walls

Australia

Ken Wallston

United States of America

Adam Walsh

Australia

Denis Walsh

United Kingdom

Henry Wamani

Norway

Joyce Wamoyi

Tanzania

Handan Wand

Australia

Gustavo Wandalsen

Brazil

JianLi Wang

Canada

Jinfeng Wang

China

Weibing Wang

China

Yanping Wang

China

Ning Wang

China

Yuying Wang

China

Yan Wang

United States of America

Duolao Wang

United Kingdom

Jian Wang

China

Samuel Wanji

Cameroon

Helen Ward

United Kingdom

Bernadette Ward

Australia

Douglas Fraser Wares

Switzerland

Nasir Warfa

United Kingdom

Richard Watt

United Kingdom

Andrea Waylen

United Kingdom

Marianne Weber

Australia

Mary Beth Weber

United States of America

Jayne Webster

United Kingdom

Niels Wedderkopp

Denmark

Xiaolin Wei

China

Anita Weidmann

United Kingdom

Olivier Weil

France

Catherine Weil-Olivier

France

Stevan Weine

United States of America

John Weinman

United Kingdom

Jennifer Weisent

United States of America

Renata Welc-Faleciak

Poland

Yvonne Wells

Australia

Tzai-Hung Wen

Taiwan

Xiaozhong Wen

United States of America

Jaroslava Wendlova

Slovakia

Guilherme Werneck

Brazil

Guido Werner

Germany

Bo Werner

Sweden

Robert West

United Kingdom

Peter J.M. Westerholm

Sweden

Klaas Westerterp

Netherlands

Kath Weston

Australia

Tjalke Westra

Netherlands

Catherine Wetmore

United States of America

Joan Wharf Higgins

Canada

Benedict Wheeler

United Kingdom

David Wheeler

United States of America

James White

United Kingdom

Dean Whitehead

New Zealand

James Whitworth

United Kingdom

Michael Whyte

United States of America

Sabine Wicker

Germany

Ellen Wiebe

Canada

Darryl Wieland

United States of America

Frank Wieringa

France

Nora Wiium

Norway

Joellen Wilbur

United States of America

Rose Wilcher

United States of America

Anna Wilkinson

United States of America

Merlin Willcox

United Kingdom

Joshua Willey

United States of America

Gary Williams

United Kingdom

Anna Williams

Australia

Emily Williams

Australia

Lauren Williams

Australia

Iestyn Williams

United Kingdom

Andrew Willis

United Kingdom

Leigh Wilson

United States of America

Abigail Wilson

United States of America

Paul M Wilson

United Kingdom

Michael Wilson

United States of America

Carl Heinz Wirsing von Koenig

Germany

Julia Wirth

United States of America

Robert Wisse

Netherlands

Viroj Wiwanitkit

Thailand

Tony Wohlers

United States of America

Mirkuzie Woldie

Ethiopia

Li Ping Wong

Malaysia

Imelda Wong

Canada

Jong Min Woo

Korea South

Caroline Wood

United Kingdom

Sara Wood

United Kingdom

James Wood

Australia

James Wood

United Kingdom

Sarah Woodhall

United Kingdom

Alistair Woodward

New Zealand

Kathleen Woolf

United States of America

Alemayehu Worku

Ethiopia

Lemu Golassa Woyessa

Ethiopia

Chiung-Jung (Jo) Wu

Australia

Zunyou Wu

China

Winfred Wu

United States of America

Sara Wuehler

Ethiopia

Blair J. Wylie

United States of America

Karen Wynter

Australia

Bo Xi

China

Huiyun Xiang

United States of America

Fang Xie

United States of America

Jian Xing

United States of America

Yin Xu

United States of America

Hairong Xu

United States of America

Jieshuang Xu

China

Guoshuang Xu

China

Derek Yach

United States of America

Rajpal Singh Yadav

Switzerland

Kapil Yadav

India

Reza Yaesoubi

United States of America

Bereket Yakob

Ethiopia

Thespina Yamanis

United States of America

Hong Yan

China

Weili Yan

China

Gonghuan Yang

China

Xiaolin Yang

Finland

Xilin Yang

China

Lin Yang

Hong Kong

Mohammed Yassin

Switzerland

Mei-Yu Yeh

Taiwan

Francis Yeji

Ghana

Edward Yelin

United States of America

Cheng-Fang Yen

Taiwan

Hyeon-woo Yim

Korea South

Solomon A Yimer

Norway

Paul Yip

Hong Kong

Yu Yizhen

China

Pekka Viljo Ylöstalo

Finland

Hua Yong

Australia

Jin-Sang Yoon

Korea South

yeong sook Yoon

Korea South

Sze Lin Yoong

Australia

Robert Young

United Kingdom

Ian Young

United Kingdom

David Young

Australia

Ilham Youssry

Egypt

Børge Ytterstad

Norway

Tao Yu

Singapore

Dongmei Yu

China

Ruby Yu

Hong Kong

Baorong Yu

China

Shumei Yun

United States of America

Basia Zaba

United Kingdom

Lydia Zablotska

United States of America

Anna Zajacova

United States of America

Rubeena Zakar

Germany

Nickolas Zaller

United States of America

Roger Zapata

Peru

Milos Zarkovic

Serbia

Donald Zeigler

United States of America

Jean-Pierre Zellweger

Switzerland

Shannon Zenk

United States of America

Chloe Zera

United States of America

Hong Zhang

United States of America

Yonghong Zhang

China

ZhiJie Zhang

China

Lei Zhang

Australia

Tuohong Zhang

China

Qingyuan Zhang

China

Jing Zhao

China

Dong Zhao

China

Wenhua Zhao

China

Xudong Zhao

China

Motao Zhu

United States of America

Xiaohui Zhuo

United States of America

Ekhard Ziegler

United States of America

Fred Zijlstra

Netherlands

Gregory Zimet

United States of America

Heidi Zinzow

United States of America

Marija Zivkovic-Gojovic

Canada

Huachun Zou

Australia

Maria Victoria Zunzunegui

Canada

Li Zuo

China

Dejan Zurovac

Kenya

Yvonne Zurynski

Australia

Virginia Zweigenthal

South Africa

## Pre-publication history

The pre-publication history for this paper can be accessed here:

http://www.biomedcentral.com/1471-2458/13/223/prepub

